# Structure, Dynamics and Implied Gating Mechanism of a Human Cyclic Nucleotide-Gated Channel

**DOI:** 10.1371/journal.pcbi.1003976

**Published:** 2014-12-04

**Authors:** Yana Gofman, Charlotta Schärfe, Debora S. Marks, Turkan Haliloglu, Nir Ben-Tal

**Affiliations:** 1 Department of Biochemistry and Molecular Biology, George S. Wise Faculty of Life Sciences, Tel-Aviv University, Tel Aviv, Israel; 2 Center for Bioinformatics, Quantitative Biology Center, and Department of Computer Science, Tübingen University, Tübingen, Germany; 3 Department of Systems Biology, Harvard University, Boston, Massachusetts, United States of America; 4 Polymer Research Centre and Chemical Engineering Department, Bogazici University, Bebek-Istanbul, Turkey; Institut Pasteur, France

## Abstract

Cyclic nucleotide-gated (CNG) ion channels are nonselective cation channels, essential for visual and olfactory sensory transduction. Although the channels include voltage-sensor domains (VSDs), their conductance is thought to be independent of the membrane potential, and their gating regulated by cytosolic cyclic nucleotide–binding domains. Mutations in these channels result in severe, degenerative retinal diseases, which remain untreatable. The lack of structural information on CNG channels has prevented mechanistic understanding of disease-causing mutations, precluded structure-based drug design, and hampered *in silico* investigation of the gating mechanism. To address this, we built a 3D model of the cone tetrameric CNG channel, based on homology to two distinct templates with known structures: the transmembrane (TM) domain of a bacterial channel, and the cyclic nucleotide-binding domain of the mouse HCN2 channel. Since the TM-domain template had low sequence-similarity to the TM domains of the CNG channels, and to reconcile conflicts between the two templates, we developed a novel, hybrid approach, combining homology modeling with evolutionary coupling constraints. Next, we used elastic network analysis of the model structure to investigate global motions of the channel and to elucidate its gating mechanism. We found the following: (i) In the main mode of motion, the TM and cytosolic domains counter-rotated around the membrane normal. We related this motion to gating, a proposition that is supported by previous experimental data, and by comparison to the known gating mechanism of the bacterial KirBac channel. (ii) The VSDs could facilitate gating (supplementing the pore gate), explaining their presence in such ‘voltage-insensitive’ channels. (iii) Our elastic network model analysis of the CNGA3 channel supports a modular model of allosteric gating, according to which protein domains are quasi-independent: they can move independently, but are coupled to each other allosterically.

## Author Summary

Cyclic nucleotide-gated (CNG) channels mediate the passage of cations through the cytoplasmic membrane. They are involved in sensory transduction and cellular development in the rod and cone photoreceptors, as well as in brain, kidney, heart and other cells, and are linked to achromatopsia and other rare genetic diseases. We used a hybrid modeling approach, combining comparative modeling and estimates of evolutionary conservation and couplings, to model the structure of a human cone CNG channel. The channel comprises a membrane domain that allows ion passage, and a regulatory cytosolic domain that binds cyclic nucleotides. The structure of each domain was modeled by homology on the basis of a suitable template. Our hybrid approach allowed us to evaluate the model structure, as well as to determine the conformations of regions where the templates overlapped and presented conflicting structural evidence. We then conducted normal mode analysis to reveal global motions of the channel. We suggest that the main mode of motion, counter-rotation of the membrane and cytosolic domains around the membrane normal, is associated with channel gating. Such rotational motion induces minimal perturbation to the lipid membrane, which could explain why the motion is observed in other types of channels.

## Introduction

Cyclic nucleotide-gated (CNG) ion channels are nonselective cation channels, essential for visual and olfactory sensory transduction in vertebrates [1]–[4]. Like other members of the voltage-gated-like ion channel superfamily [2], the CNG channels are composed of four (identical or similar) monomers, each containing six transmembrane (TM) helices (referred to as S1–S6) [1], [3]. The first four TM helices in each monomer (S1–S4) form a voltage-sensor domain (VSD); the last two helices (S5 and S6, connected by the P-loop) of the four subunits assemble jointly to form the central pore. In spite of the presence of the VSD, CNG channels display very little voltage-dependent activity [1]–[4]. Rather, the channel is gated by cyclic nucleotide binding to a cytosolic cyclic nucleotide–binding domain (CNBD), connected by a so-called C-linker to the C-terminus of the S6 helix [1]–[4].

In vertebrates, the six known members of the CNG channel family, classified into A and B subtypes, can coassemble in several combinations to produce functional heterotetrameric channels [1]–[6]. In cone photoreceptors, functional tetrameric CNG channels are composed of two CNGA3 and two CNGB3 subunits, with alike subunits positioned next to each other [7] (see also a recent work by Ding and colleagues suggesting a composition of three CNGA3 subunits and one CNGB3 subunit in a cone channel [8]). Binding of cyclic guanosine monophosphate (cGMP) to CNBD controls the activity of the cone channels [1], [3], [6]. The cone system confers color vision; about 70 mutations in the CNGA3 and CNGB3 have been associated with achromatopsia, characterized by color blindness, photophobia, nystagmus and impaired visual acuity [3]. However, most of these mutations have been observed only in isolated cases, and even among the more commonly-observed mutations the precise mechanisms causing diseases are unknown (Tables S1 and S2) [3], [9], thus hindering drug-discovery efforts. These challenges are further complicated by the poor understanding of the gating mechanism of the CNG channel, despite the available structural information on the membrane domain of a bacterial homolog [10] and on the regulatory cytosolic domain of both CNG and closely related hyperpolarization-activated cyclic nucleotide-gated (HCN) channels from various organisms [11]–[20].

Several different models have been proposed to describe the gating process of CNG channels (reviewed in [5], [21]–[23]). The models differ from one another in the minimum number of bound nucleotides required for activation, the cooperativity among the binding sites, and the number of open and closed states. The simplest model, known as the sequential model, suggests that three ligand molecules bind to the closed channel, and that the binding of a fourth molecule causes the transition to the open state [24]. The sequential model does not account for the experimentally confirmed opening of unliganded or partially-liganded CNG channels and is therefore insufficient [25]. The classical Monod, Wyman, and Changeux (MWC) model [26], initially aimed at describing cooperative properties of bacterial regulatory enzymes and hemoglobin, postulates that the channel can open with any number of bound ligands, i.e., 0 to 4; the higher the number of bound ligands, the more favorable the transition from the closed to the open state [5]. Although the MWC model accurately describes some features of CNG channels, it does not account for the existence of sub-conductance states, allowing only one open state [22]. A third model describes the tetramer as a dimer of dimers; each dimer acts as an MWC unit, and the monomers within the dimer display cooperativity [27]. In order for the channel to open, both dimers must be activated independently (no cooperativity between the dimers). The last model, the modular gating model, defines modules within the channel: the VSD, the pore, the C-linker and the CNBD [28]. Each module can independently switch between two possible conformations: the VSD and the C-linker can be either resting or activated; the pore can be closed or open; and the CNBD can be ligand-free (apo) or –bound (holo). Yet, the modules are coupled to each other, so that the state of each module can affect the states of other modules.

In order to elucidate the channel gating mechanism, we built 3D models of the tetrameric CNG channel in its resting state, with the CNBDs in apo- and holo-conformations. We built the homology model from two distinct templates—one corresponding to the TM domain and the other corresponding to the cytosolic domain—and evaluated the model structure on the basis of its evolutionary conservation profile [29]. However, because the modeling process was complicated by the use of two distinct templates, as well as by low sequence similarity between the queries and the template for the TM domain, we also evaluated the model structure using EVcouplings, a recently-developed methodology that identifies evolutionarily coupled residues from sequence variation data [30], [31]. As evolutionarily coupled residues often interact physically in the 3D space of a protein [32], this and similar methods (reviewed in [33], [34]) have been successfully applied to predict structures of membrane and soluble proteins, as well as to detect residues participating in conformational changes and protein-protein interactions. Here we used evolutionary couplings (i) to evaluate our model structure of the cone channel and (ii) to select the best conformations in ambiguous regions of the model and in regions in which the two templates yielded overlapping, conflicting predictions.

To analyze the channel dynamics, we used coarse-grained elastic network models [35]–[37]. In the channel's slowest, dominant mode of motion, the TM and cytosolic domains rotate around the membrane normal in opposite directions. We related this mode of motion to gating, a proposition supported by experimental evidence [38], [39], as well as by comparison to the gating mechanism of the bacterial KirBac channel. KirBac channels resemble CNG channels in the architecture of the pore domain and in the function of the cytoplasmic domain. Additionally, investigation of the next-slowest modes of motion revealed that the TM and the cytosolic domains fluctuate alternatively in each mode, with cooperativity between the mobile and immobile domains. This observation is consistent with the modular gating model of the cone channel [28].

## Results

### The model structure displays typical conservation and hydrophobicity profiles and is compatible with experimental results in the homologous CNGA1 protein

**Figure 1:**
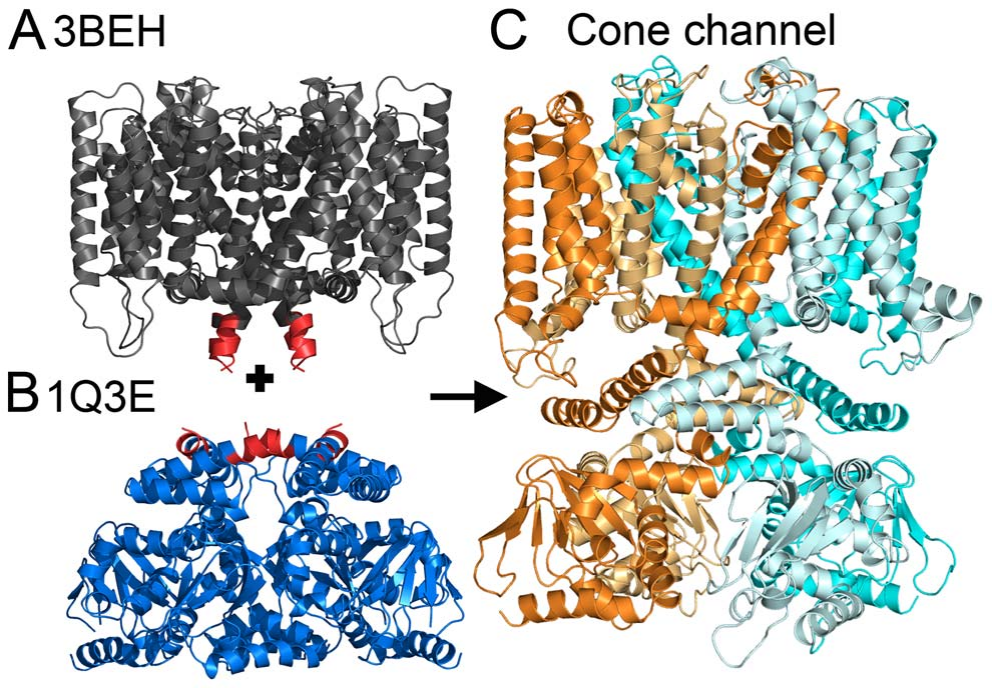
Modeling of the full-length cone channel in its resting state, on the basis of two separate templates. (A) Side view of the TM region of the bacterial channel MlotiK1 in a closed state, PDB entry 3BEH [10]. This structure was the template for modeling the TM region of the cone channel. (B) Side view of the mouse HCN2 CNBDs in a resting state, PDB entry 1Q3E [15]. This structure served as a template for the cytosolic domain of the cone channel. (A, B) The regions of conflict between the templates are in red. (C) Side view of the resultant model structure of the human cone channel, shown in cartoon representation. CNGA3 subunits are colored cyan (light and dark); CNGB3 subunits are colored orange (light and dark).

**Figure 2:**
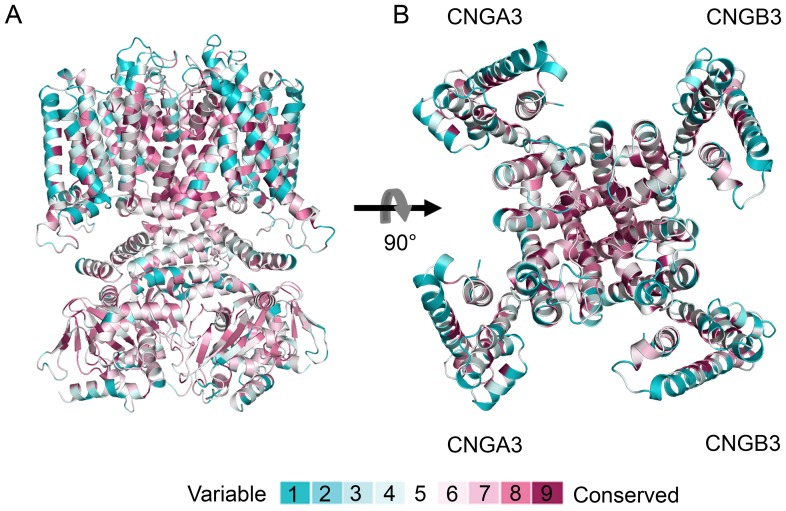
The evolutionary conservation profile supports the model structure. The channel is colored by conservation grades according to the color-coding bar, with variable-through-conserved corresponding to turquoise-through-maroon. Overall, the variable residues are peripheral, whereas the conserved residues are in the structural core and channel pore. (A) Side view of the model structure in cartoon representation; the CNBDs are in their holo-state. (B) Extracellular view of the TM domain of the model structure. The proposed locations of the two CNGA3 and two CNGB3 subunits are marked [7].

We modeled by homology the 3D structure of the cone CNG channel using two distinct templates: the TM region structure of the bacterial channel MlotiK1, and the cytosolic domain structure of the mouse HCN2 channel (Fig. 1). We projected ConSurf [40] conservation scores of the CNGA3 and CNGB3 subunits, composing the cone channel, onto the resultant model to evaluate it (Fig. 2). The evolutionary conservation profile has previously been demonstrated to be a valuable tool for assessing the quality of model structures; the assessment approach is based on the observation that the protein core is typically conserved, whereas residues that face the surroundings, either lipids or water, are variable [29]. Our model structure was compatible with the anticipated evolutionary profile (Fig. 2). The model structure of the TM domain was also compatible with the expected hydrophobicity profile: hydrophobic residues were exposed to the lipid, and charged and polar amino acids were buried in the protein core or located in the loop regions (Figure S1). The model correlates well with the experimental data available for the closely homologous bovine channel CNGA1 (Text S1; Figures S2 and S3). Furthermore, it suggests molecular interpretations for the effects of known clinical mutations (Text S1; Tables S1 and S2; Figure S4).

### The model structure is mostly compatible with predicted evolutionary couplings

**Figure 3:**
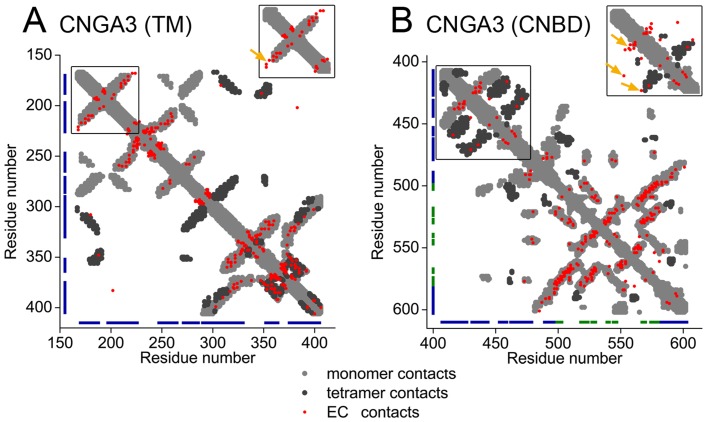
Evolutionary coupling (EC) calculations support the model. Contact maps of top-ranked predicted ECs (red) overlaid on monomer (light grey) and intermonomer (dark grey) contacts from (A) the model structure of the TM domain of CNGA3; (B) the model structure of the cytosolic domain of CNGA3. The insets show the contact maps of alternative CNGA3 model structures; the orange arrows point to evolutionary couplings between amino acid pairs that are not in contact in the alternative models, but are in contact in our final model. Clearly, the overlay of the ECs with the contact map is far better for the chosen model structure than for the alternatives.

We further evaluated the predicted model structure of the CNGA3 monomer and tetramer using evolutionary couplings calculated separately for the TM domain and for the cytosolic domain. To this end, we used the EVcouplings algorithm [30], [31]. For each domain we carried out the comparison using the 2L/3 residues with the greatest coupling strength, where L is the sequence length; L = 240 in the TM region and L = 197 in the cytoplasmic region; see Methods. In both domains the overlay of the calculated evolutionary couplings and the contacts derived from the model structure was remarkable (Fig. 3): among the residue pairs used in the evaluations of the TM and cytosolic domains, 75% and 92%, respectively, were in contact. A control calculation using the templates revealed similar ratios of contacting evolutionary couplings in the two domains (Figure S5). A more detailed analysis of the couplings detected between residues that were not in contact according to the model structure (or the templates) suggested that most of these are related to flexibility in the loop regions (Text S1).

### Evolutionary couplings support the model structure in regions with conflicting structural data

Overall, our model structure was compatible with the anticipated hydrophobicity (Figure S1) and evolutionary (Fig. 2) patterns, as well as with mappings of evolutionary couplings (Fig. 3). However, we considered alternative conformations for two specific regions in the model, i.e., helix S1 of the TM domain (Figure S6) and the S6-C-linker interface of the cytosolic domain (Fig. 1). First, secondary structure prediction methods (Figures S6A and S6B), in addition to the hydrophobicity profile of the homologs in the region (Figure S6D), pointed to two main alternatives for the boundaries of helix S1: 170–186, used in the original model, vs. 174–190 (Figure S6D). Second, for the region connecting helix S6 and the C-linker, either of the two templates could have been used (i.e., either the TM domain of bacterial MlotiK1 channel or the cytosolic domain of the mouse HCN2 channel), and we based our model structure on the mammalian template. However, we considered an alternative model structure, in which the conformation of this region was based on the bacterial template (Figs. 1A and 1B). Overall, we constructed two alternative model structures of the CNG channel (in addition to the original): one with different boundaries for the S1 helix, and another one with a different conformation of the S6-C-linker interface. We correlated the overlay of the contacts in these models with the evolutionary couplings and concluded that the original model agrees with the data better than either alternative (Fig. 3, the insets).

### Equilibrium dynamics of the CNGA3 channel

We used coarse-grained elastic network models to investigate global motions of the cone channel. We chose this methodology because it is insensitive to the atomic details of the (imprecise) model structure and is capable of exploring large-scale motions, related to channel gating and inactivation [37], [41]. For simplicity and to facilitate more convenient representation of the data, we focused on a symmetric tetrameric channel composed of four identical CNGA3 subunits. The results obtained for the structure, modeled based on CNBD templates in their apo- and holo-states, were, in essence, identical, and thus we only describe the results obtained for the apo-state.

**Figure 4:**
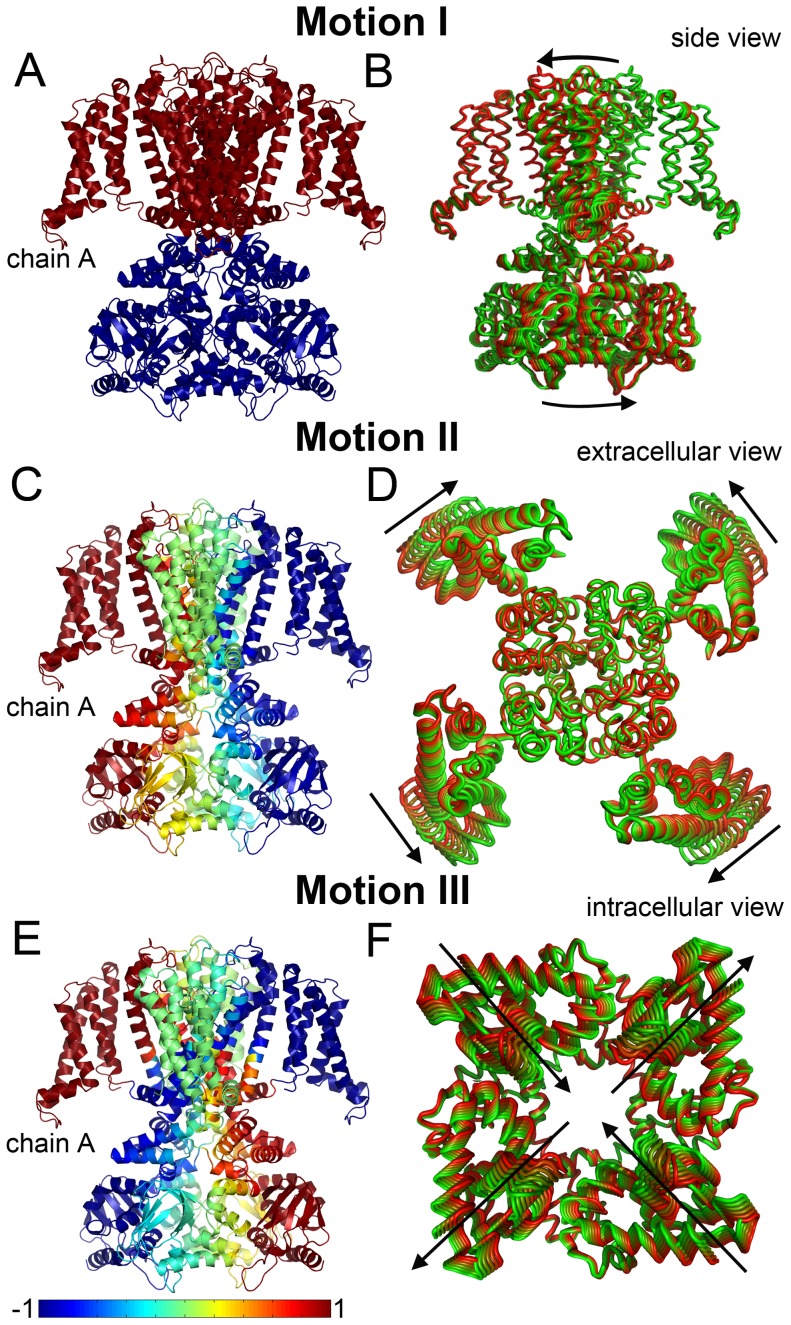
The three principal motions. The cross-correlations between the residues in each motion type are shown on the left panels (A, C, E); the channel is colored according to the correlation of the VSD of chain A with the other residues. The corresponding motions are presented on the right panels (B, D, F). Snapshots are colored according to the direction of motion, ranging from green to red. (A, C, E) In each panel, chain A represents an arbitrary CNGA3 chain (the channel comprises four identical chains). The magnitude of positive and negative correlations between the fluctuations of the residues is color-coded according to the blue-to-red scale at the bottom of the picture. Positive correlation indicates motion of two residues in the same direction, while negative correlation indicates motion in opposite directions. In panel A, the arrows indicate the location of the pivot points of the rotational motion at the termini of the S6 helices, i.e., the border between the dynamics units (See Discussion). The CNBDs in panel D and the TM domain in panel F are omitted for clarity.

A complete description of the results is provided in Text S1. Briefly, the channel dynamics are dominated by three types of motion. In motion I the channel is divided into two dynamic units: the TM domain and the cytosolic domain (Fig. 4A). The two domains rotate around the membrane normal in opposite directions (Fig. 4B). In motion II the VSDs are swinging, while the pore domain, as well as the C-linkers and the CNBDs, are essentially stationary (Fig. 4D and Figure S7B). The fluctuations of the VSDs are positively correlated with those of the C-linker and the CNBD of the same subunit, and with those of the pore region of the adjacent subunit (Fig. 4C). In motion III the pairs of diagonal CNBDs alternately move towards and away from each other (Fig. 4F). The fluctuations of the VSDs are positively correlated with those of the CNBD, C-linker and pore region of the adjacent subunits (Fig. 4E).

The analysis also shows that, in essence, all the motions, except motion I, can be categorized into modes in which only the TM domain is fluctuating (as in motion II) or modes in which only the cytosolic domain fluctuates (as in motion III). Thus, each domain is mobile individually. However, there is cooperativity between the domains, which suggests that they affect each other (Figs. 4C and 4E). This observation corroborates the modular gating model of the CNG channels, which postulates that the domains (modules) can undergo conformational changes individually, but that the state of each module affects the states of other modules, i.e., quasi-independent dynamic units [28].

In order to examine the effect of the VSDs on channel dynamics, we performed elastic networks analysis of a variant of the channel in which the VSDs were removed (Figure S8). The dynamics remained, in essence, the same, aside from the motions that directly involve the VSDs.

## Discussion

We presented a model structure of a human CNG channel, built using a unique computational protocol that includes homology modeling, as well as evolutionary data from conservation and couplings analysis. The model correlates well with mutagenesis and clinical data. Elastic network analyses of the model-structure enable us to provide concrete suggestions concerning the gating mechanism.

### Rotational motion and gating

**Figure 5:**
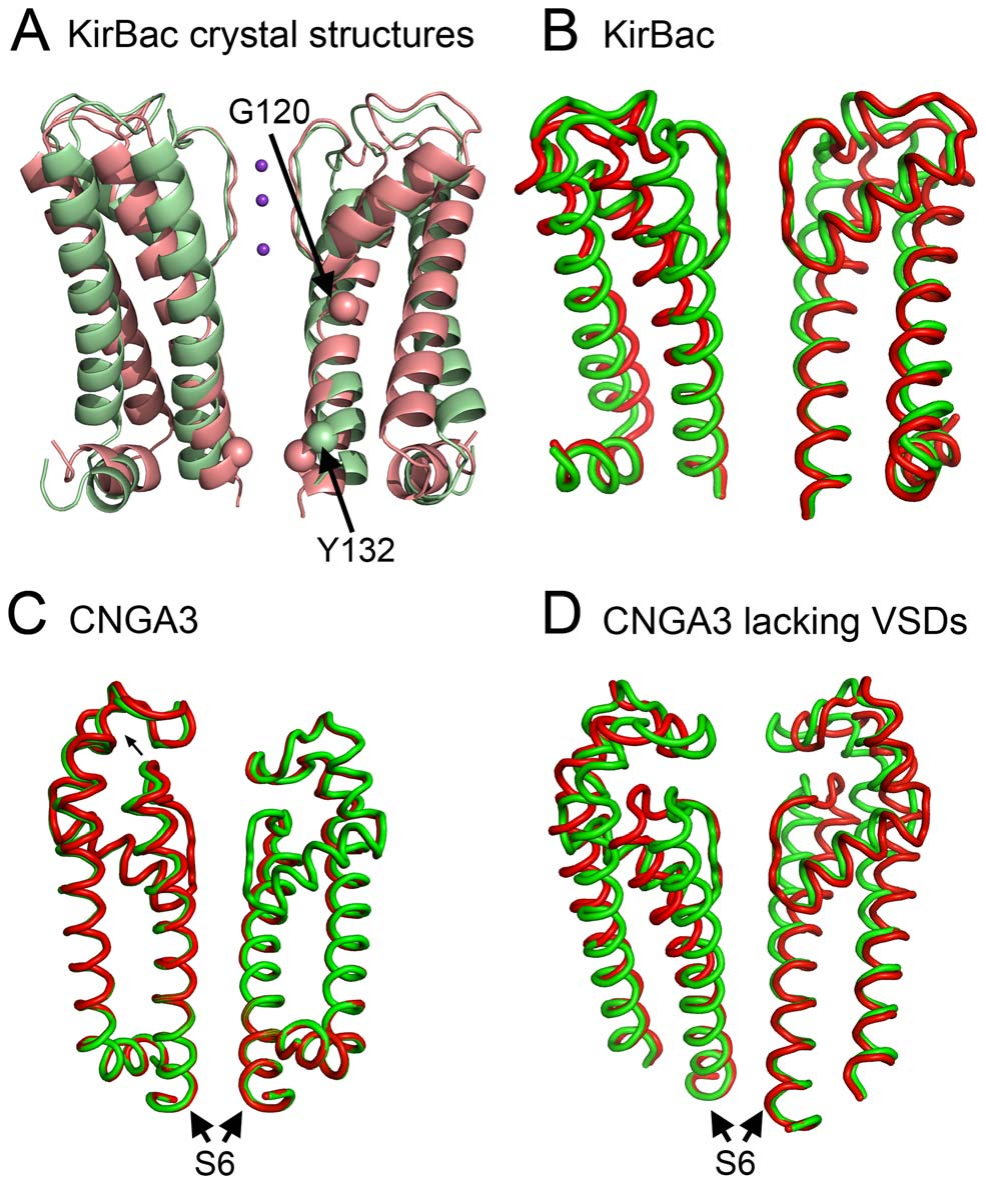
Motion I of CNGA3 appears to describe channel gating. For clarity, only helices S5 and S6 (or corresponding KirBac helices) of two juxtaposed subunits are shown in each panel. (A, B) The similarity between the predicted conformations in panel B and the crystal structures in panel A is apparent, verifying the relation between these conformations and channel gating. (A) Side view of the KirBac3.1 crystal structures in open (pale green, PDB entry 3ZRS [43]) and closed (pale red, PDB entry 2WLJ [44]) states. The α-carbons of the two gate-residues, namely G120 and Y132, are shown as a space-filling model. (B–D) The edge conformations of KirBac3.1 (B), CNGA3 (C) and CNGA3 lacking the VSDs (D), as predicted by the elastic network models in the slowest mode of motion. The two edge conformations are shown in red and green. (C) The CNGA3 conformations resemble the conformations predicted for the KirBac channel (panel B), but the pore region is rigid. (D) The CNGA3 without VSD conformations are identical to the KirBac conformations (panel B).

Motion I of the CNGA3 channel is a rotational, iris-like opening (Figs. 4A and 4B). This motion is unique in that it is associated with the only (non-degenerate) slow mode that manifests cooperativity among all subunits and allows symmetry-preserving conformational transition. Here we compare this motion with the gating motion in KirBac channels, prokaryotic homologs of mammalian inwardly rectifying potassium channels. KirBac channels share the architecture of the pore domain with other members of the voltage-gated-like ion channel superfamily, but they lack the VSD. Similarly to CNG channels, KirBac channels feature a cytoplasmic regulatory domain [42]. Recent crystal structures of the KirBac3.1 channel in the open and closed states (Fig. 5A) revealed its gating mechanism: upon activation, the TM and cytoplasmic domains of KirBac3.1 rotate in opposite directions around the membrane normal [43], [44]. For comparison, we performed elastic network analysis of the KirBac3.1 channel in its closed state.

The slowest mode of motion of the KirBac3.1 channel displayed rotational movement of the TM and cytoplasmic domains, which resembled the shift between the open and closed states captured in the crystal structures, i.e., the conformational change that occurs upon gating (Figs. 5A and 5B) [43]. Because of the analogy between this motion and motion I of the CNG channel, we associate the latter motion with gating as well (Fig. 5). Indeed, calculations using the edge conformations of the motion and the HOLE software [45] show that the counter-clockwise rotation of the TM domain (and the joint clockwise rotation of the cytoplasmic domain) leads to pore opening (Figure S9).

The idea of a rotational motion related to the gating of CNG channels is not new [38], [39]. Based on existing experimental evidence, Flynn and colleagues have associated a clockwise rotation of the C-linkers with channel gating (reviewed in [39]). The authors also indicated that a rotational gating motion requires definition of a ‘pivot point’, the residues on the two sides of which rotate in opposite directions. They proposed that in CNG channels the pivot point is located at the top of the S6 helices. Indeed, our elastic network analysis shows clockwise rotation of the cytosolic domain, which is coupled to counterclockwise rotation of the TM domain (Fig. 4A), an observation compatible with their proposition and with the gating mechanism of the KirBac channels [43]. However, our analysis indicates that the pivot point is located at the C-termini (rather than the top) of the S6 helices (Fig. 4A); it corresponds to the hinge in motion I around residues 401–407 ().

Although CNG and KirBac channels display similar rotational motion upon gating, there is an important difference between the two channels. KirBac channels contain two activation gates: one in the center of the S6 helix (near the P-loop) and one at the C-termini of the S6 helices (Fig. 5A) [43]. In contrast, CNG channels contain only a single gate, which is located in the pore region [21], [39]. We propose that in the absence of a second gate in the CNG channels, the VSD provides an additional means of gating regulation. Our conclusion derives from a close investigation of rotational motion I of the CNGA3 channel in the presence (Fig. 5C) and absence (Fig. 5D) of the VSDs. In the presence of the VSDs (Fig. 5C), the fluctuations of the N-termini of the S6 helices are smaller in magnitude than they are in the absence of these domains (Fig. 5D). In other words, the VSD stiffens the pore region, so that the fluctuations in the gate region are limited. This could explain why KirBac channels, lacking the VSD, have an extra gate at the C-termini of the S6 helices [43], whereas CNG channels, which do contain the VSD, have only one gate.

The idea that rotational motion can facilitate gating has been proposed for other ion channels from various families, both voltage-dependent and -independent [46]–[52]. This motion appears to have minimal effect on the channel-membrane interface, and causes minimal perturbation of the lipid membrane. Thus, it is possible that many more ion channels share a similar rotational gating mechanism.

### Limitations of the study

The approach used to predict the structure of the cone channel has certain drawbacks. First, the TM region was derived from the structure of a distant homolog, with ∼10% sequence identity between query and template. This may have led to inaccuracies in the model structure due to errors in the alignment of the query and template sequences, as well as structural changes along the course of evolution. However, the compatibility of the model structure with the expected evolutionary and hydrophobicity patterns, evolutionary couplings and the available experimental data is encouraging (Figs. 2, 3 and Figures S1-S3). Second, our model structure of the cone channel was derived from two distinct templates. Therefore, the interface connecting these templates in the model, i.e., the S6-C-linker region, might be inaccurate. However, we have explored alternative conformations of the region, and the evolutionary couplings agreed best with our model structure (Fig. 3B). Third, the loop regions in any model structure are expected to be imprecise [53]. Reassuringly, the exact loop conformation that was chosen had little effect on the results of our elastic network analysis. The fact that the results are insensitive to minor structural changes suggests that other channels of the CNG family might share dynamics similar to those of CNGA3. Lastly, the fact that the cytosolic domain was modeled on the basis of the crystal structure of the corresponding domain of an HCN channel could also be problematic, as the resemblance of the CNG and the HCN channels in this region has previously been called into question, owing to conflicting experimental evidence [54]. That the evolutionary couplings in the cytosolic domain of the human CNGA3 channel and mouse HCN2 channel displayed similar patterns suggests that they do share the same conformation in this region (Fig. 3B and Figure S5B).

Given the weaknesses of the presented model structure, it is quite natural to study the channel dynamics using elastic network analysis, an approach that relies on a simplified representation of the channel structure, comprising α-carbons connected by Hookean springs of identical force constant [35], [55]–[57]. That is, the network models do not depend on the identity of the amino acids and the specificity of interactions between them. Moreover, the coarse-grained network models do not depend on the atomic details of the structure and can tolerate some variations in topology [37], [41]. In addition, elastic network models are capable of capturing large-scale conformational changes, including changes that are dependent on external stimuli, such as voltage or ligand binding. This is because the intrinsic modes of motion of a protein are determined by its architecture only, and the elastic network models can predict these modes solely from the structure, regardless of the environment (water or membrane) [37]. Whereas the environment can have an impact on the magnitude of the global motions and on local interactions, it does not usually determine their direction and nature [37]. Indeed, in previous studies, slow modes of motion have been shown to describe functional conformational changes in proteins, including membrane channels and transporters [35]–[37], [41]. Finally, the strongest support for the suitability of elastic network models as an approach for detecting functionally important motions in CNG channels comes from calculations we conducted with the KirBac3.1 channel: The dominant motions we detected for the CNG channels were very similar to motions calculated for the KirBac3.1 channel, inferred to correspond to gating motions, according to the known crystal structures of the channel in the open and closed states (Fig. 5).

Evolutionary couplings have been successfully used to predict the structures of membrane and soluble proteins (reviewed in [33], [34]), but our preliminary attempt to predict the structure of the CNG channel using evolutionary couplings alone was unsuccessful. This is perhaps because of the large number of inter-subunit contacts in the unique architecture of the tetrameric CNG channel; it is difficult to discriminate between couplings associated with the intra- and inter-subunit contacts. Instead, we used homology modeling to derive our model structure, and relied on evolutionary couplings to validate the model and to distinguish between possible conformations in regions of conflicting structural evidence. This is somewhat related to the approach used in reference [58].

In some cases, evolutionary couplings may reflect protein conformational changes, as demonstrated for several TM transporters [30]. However, our evolutionary couplings map provided no clues as to the channel's conformational changes. We suggest that the reason is that, in contrast to the major conformational changes observed in transporters, conformational changes in channels, including the rotational motion described above, are minor and have little effect on residue contacts.

While this paper was in review, a homology model of the TM domain of the canine CNGA3 channel was published [59]. The model, obtained based on a chimeric Kv1.2/2.1 structural template, corresponds to an open state of the channel, while our model structure represents a closed conformation. The models are based on different templates, and they also differ from each other in the boundaries of the TM helices (Figure S6A); most differences could perhaps be attributed to the conformational changes upon channel opening/closure.

### Conclusions

We presented a 3D model structure of the heterotetrameric human cone channel, composed of CNGA3 and CNGB3 subunits, performed elastic network model analysis of the (equivalent homotetrameric CNGA3) channel, and obtained a mechanistic view of its gating. The following are three ‘take-home messages’:

1. We suggest that the slowest mode of motion, describing counter-rotations of the TM and cytosolic domains around the membrane normal, depicts channel gating. A similar gating mechanism has been proposed for other ion channels.
2. The presence of the VSD in the voltage-insensitive CNG channels is a long-standing conundrum [10]. Recently, it was shown that the domain affects trafficking [60], but one could argue that from a parsimonious point of view it seems unlikely that a whole domain would be preserved in evolution in a case where a short peptide would do. Locations of high-frequency disease-associated mutations (Text S1 “Mapping disease-causing mutations on the model structure”) and normal mode calculations together suggest that the VSDs may also play a role in gating, supplementing the pore gate.
3. Among the allosteric models of gating in CNG channels, the elastic network model analysis of the CNGA3 channel supports the modular gating variant. According to this model, autonomous domains that can move independently are coupled to each other such that conformational changes in one may allosterically propagate within the tetrameric channel structure [5], [28].

## Methods

### Modeling of the CNGA3 and CNGB3 subunits

A CNG channel subunit consists of a TM domain and a cytosolic domain comprising a CNBD and a C-linker. In the absence of a high-resolution structure of an intact subunit, we modeled the cone channel on the basis of existing structures corresponding to the individual domains. CNG and HCN channels are closely related in structure [5]; thus, HCN structures can serve as templates for modeling CNGs. A variety of high-resolution structures of mammalian cytosolic domains from HCN channels are available [15]–[20]. As a template for the apo-state, we used the mouse HCN2 channel (Protein Data Bank (PDB) entry 3FFQ [18]), the only available structure of a mammal cytosolic domain in an apo-state. As a template for the holo-state, we used the mouse HCN2 channel (PDB entry 1Q3E [15]), the only available structure of the cytosolic domain in complex with cGMP. Pairwise alignments between the queries and templates were needed for the modeling. The CNBD of the mouse HCN2 channel shares sequence identities of 33% and 29% with the cytosolic domains of CNGA3 and of CNGB3, respectively. When a query and a template display sequence identity of 30% or more, a model structure can be constructed on the basis of a simple pairwise alignment [53]. Still, extraction of the pairwise alignment from a multiple-sequence alignment can improve the model accuracy [53]. We searched for the homologs of the mouse HCN2 channel in the CleanUniProt database [61] and aligned their sequences using the MUSCLE program [62]. Both subunits, CNGA3 and CNGB3, were detected as homologs, and their alignments with the sequence of the mouse HCN2 channel were extracted from the multiple sequence alignment (Figure S10).

The crystal structure of the bacterial MlotiK1 channel in its closed state (PDB entry 3BEH [10]) served as a template for modeling the TM domains of CNGA3 and CNGB3. The sequence identities between the TM domain of MlotK1 and those of CNGA3 and CNGB3 were only 10% and 12.5%, respectively. Thus, aligning both queries to the template was challenging. In cases of such low query-template similarity it is recommended to use a variety of tools in order to produce a reliable pairwise alignment [53]. We exploited the secondary structure prediction algorithm PsiPred [63], several methods for the identification of TM segments, namely MEMSAT [64] and HMMTOP [65], a methodology for profile-to-profile alignment HHpred [66], and the FFAS03 server [67] for both profile-to-profile alignment and fold-recognition (Figure S6). Additionally, we created a multiple sequence alignment of 101 homologs that included the sequences of the queries and the template. To this end, we searched for MlotK1 homologs in the SwissProt database [68] using CS-BLAST [69]; 3 iterations were performed with maximal e-value of 10^-5^. The collected homologs were aligned using the MUSCLE program [62]. The final alignment between the queries and template took into account the outputs of all methodologies used (Figure S6). For the most part, in spite of the high sequence diversity, there was consensus in the hydrophobicity profiles of the homologs in the TM helices (e.g., Figure S6E), excluding S1. Because of the observed deviations in the predicted boundaries of the S1 segment, as well as the diversity of the hydrophobicity profiles of the homologs in the region, we considered also an alternative location of S1 in the sequence (Figure S6). Compared with this alternative, the final model was in better agreement with the evolutionary and hydrophobicity profiles, and with the evolutionary couplings. Nevertheless, our confidence regarding the boundaries of the S1 segment is lower compared with the other TM segments.

The templates for the TM (PDB entry 3BEH) and cytosolic domains (PDB entry 1Q3E) contain overlapping regions i.e., regions corresponding to the same amino acid segment in the queries: residues 217–223 of PDB entry 3BEH and residues 443–449 of PDB entry 1Q3E, corresponding to residues 407–413 in CNGA3 and residues 449–455 in CNGB3. This region corresponds to the interface between the S6 helix in the TM domain and the C-linker, and its orientation differs greatly between the templates (Fig. 1). As bacterial cytosolic domains do not include C-linkers [11], we chose to model the region of conflict according to the template of mammalian origin, i.e., PDB entry 1Q3E, covering the cytosolic domain. The initial 3D models of the CNGA3 and CNGB3 subunits were constructed using version v9.10 of the MODELLER software [70].

Long loops, i.e., the loops connecting S1–S2, S2–S3 and S5-P-loop, were refined with the Rosetta loop modeling application [71], [72]. Rosetta created 1,000 decoys for each loop. The decoys were then evaluated using the ConQuass method [29]. ConQuass is based on the anticipation that evolutionarily conserved amino acids are buried in the protein and that variable residues are exposed to the environment, and assigns scores to the decoys based on the degree to which they adhere to this pattern. In each of the three refined loops the decoy with the best ConQuass score was chosen for the final model. Evolutionary conservation profiles were calculated as described below.

For comparison, we also constructed a model structure of the CNG channel in which the S6-C-linker interface was modeled according to the bacterial template, i.e., PDB entry 3BEH, covering the TM domain. Overall, we constructed two alternative model structures of the CNG channel (in addition to the original): one with different boundaries of the S1 helix, and another one with a different conformation of the S6-C-linker interface.

### Calculation of ConSurf conservation profile

We used CS-BLAST [69] to collect homologous sequences of CNGA3 and of CNGB3 from the CleanUniProt database [61]. Redundant (>99% sequence identity) and fragmented sequences were discarded. We aligned the sequences (183 and 223 amino acids for CNGA3 and CNGB3, respectively) using the MUSCLE program [62] and then calculated the conservation profiles using the ConSurf web-server [40].

### Calculation of evolutionary couplings

A reliable calculation of evolutionary couplings requires a particularly large collection of homologous sequences [33]. We failed to find a sufficient number of CNGA3 homologs that included both the TM and CNB domains, and conducted evolutionary couplings calculations for each domain separately. Namely, we built a multiple sequence alignment for the TM domain, i.e., residues 161–410, and another alignment for the C-linker with the CNBD, i.e., residues 410–610. To this effect, we used the JackHMMer software [73] (5 iterations) to search for similar sequences against the Uniprot database [74] using the range 10^−15^–10^−20^ of e-values. These e-values yielded the largest numbers of aligned sequences while maintaining coverage of the input sequence. The final alignments covering the TM and cytosolic domains contained 6,439 and 7,203 sequences, respectively. Although proteins from families such as voltage-gated potassium channels can be aligned to the CNG channels at a high e-value threshold, these families were excluded, as they introduced many insertions and deletions. Importantly, this means that strong evolutionary couplings deduced from alignment of the VSD domain are not a consequence of inclusion of these known voltage-gated channels. Redundant (>90% sequence identity) and fragmented sequences were discarded. Evolutionary couplings were then calculated using the EVcouplings webserver (www.evfold.org) as described previously [31], [33]; covariation information was inferred using the plmDCA algorithm (pseudolikelihood maximization for Potts models with direct coupling analysis) [75]. All columns in the alignments containing gaps of less than 80% were considered informative for the calculation. The evolutionary couplings were ranked according to their coupling strength; for each domain, we took the top 2/3 L strongest evolutionary couplings (L = sequence length; L = 240 in the TM domain and L = 197 in the cytoplasmic domain) and correlated them to the contacts in the CNGA3 model structure.

For comparison, we also calculated evolutionary couplings for the template sequences of the bacterial MlotiK1 and mouse HCN2 using the same protocol. We compared the results to the contact maps deduced from the crystal structures using distance cutoffs between 10 and 15 Å; residue pairs with α-carbons below the cutoff were considered in contact. The results were qualitatively the same for all cutoff points selected. In our analysis of the model structure, we used the results based on a distance cutoff of 12 Å, reproducing over 95% of the detected evolutionary couplings.

### Elastic network models

We analyzed the model structure of the homotetrameric CNGA3 channel using two elastic network models, namely, the Gaussian network model (GNM) and the anisotropic network model (ANM). We focused on the homotetrameric channel for simplicity. The methodology of both elastic network models has been described previously [35], [55]–[57]; here we give a short summary.

GNM calculations use a simplified representation of the protein, in which the structure is reduced to α-carbon atoms and is treated as an elastic network of nodes connected by hookean springs of uniform force constant *γ*. Two nodes *i* and *j* are assumed to display Gaussian fluctuations around their equilibrium positions if the distance between them is below the (commonly used) cutoff of 10 Å. The inter-node contacts are then defined by an *N*×*N* Kirchhoff matrix ***Γ***, where *N* is the number of amino acids in the protein. The correlation between the fluctuations of two nodes *i* and *j*, Δ**R** *_i_* and Δ**R** *_j_*, respectively, are calculated as follows:
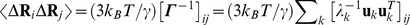
(1)where **u** *_k_* and *λ_k_* are, respectively, the *k*-th eigenvector and *k*-th eigenvalue of ***Γ***, *k_B_* is the Boltzmann constant, and *T* is the absolute temperature; *k_B_T*/*γ* was taken as 1 Å^2^. The summation is performed over all (*k* = 1 to *N*-1) non-zero eigenvalues. Overall, Eq. 1 predicts the mean-square displacement of each residue (node) when *i* = *j*, and when *i* ≠ *j* it predicts the correlations between the fluctuations of residues *i* and *j* as a superimposition of *N*-1 eigenmodes. *λ_k_* is proportional to the *k*-th mode frequency, the inverse of which gives the relative contribution of this mode to the protein's overall structural motion. The minima in the obtained fluctuation profile for a given mode suggest possible hinge points that coordinate the cooperative motions between mobile structural elements in this mode.

In contrast to isotropic GNM, ANM determines the direction of fluctuations. Here ***Γ*** is replaced by the 3*N*×3*N* Hessian matrix ***H***, the elements of which are the second derivatives of the inter-node potential described by Eq. 1, with a (commonly used) cutoff of 15 Å. The first 6 modes are zero eigenmodes, corresponding to the rigid-body rotations and translations of the protein [37], and the correlation between Δ**R** *_i_* and Δ**R** *_j_* is decomposed into 3*N*-6 modes and calculated as follows:
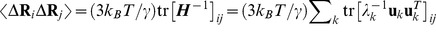
(2)where tr[H^-1^]*_ij_* is the trace of the *ij*-th submatrix [**H** ^−1^]*_ij_* of **H** ^−1^. The summation is performed over all (*k* = 1 to 3*N* - 6) non-zero eigenvalues. The eigenvectors allow us to identify alternative conformations sampled by the individual modes, simply by adding/subtracting the eigenvectors to/from the equilibrium position in the respective modes. Thus, being an anisotropic model, ANM provides information on the directions of the motions in 3D, while GNM is more realistic with respect to the mean-square fluctuations and the correlations between fluctuations [37].

Several studies have demonstrated that the first few slowest GNM modes are implicated in protein function [36], [37]. Therefore, we focused on the six GNM modes identified as slowest on the basis of the distribution of the eigenvalues; these modes were responsible for approximately 40% of the overall motion of the channel (Figure S11). The superimposition of the residues' mean-square displacement predicted by GNM and ANM revealed the correspondence between the two elastic network models. Thus, using ANM, we were able to determine the direction of fluctuations characterized by GNM.

## Supporting Information

Text S1 The file contains the following sections: “Model structure is compatible with experimental data available for the homologous CNGA1 protein”, “Mapping disease-causing mutations on the model structure”, “Evolutionary couplings between amino acids that are not in contact in the model structure”, “Equilibrium dynamics of the CNGA3 channel” and “Supplementary references”.
(PDF)

Figure S1 The hydrophobicity pattern of the model structure of the TM domain of the human cone channel from side (A) and extracellular (B) views. The model structure is colored according to the color-coding bar, with blue-through-yellow corresponding to hydrophilic-through-hydrophobic amino acids according to the Kessel/Ben-Tal hydrophobicity scale [76]. The structure displays a typical hydrophobicity pattern, with hydrophobic residues exposed to the lipid, and charged and polar amino acids buried in the protein core or located in the extramembrane regions.
(TIF)

Figure S2 The model structure of the TM domain of the human cone channel is in agreement with experimental data. (A) Intracellular view of the pore region of the channel in cartoon representation. The channel is colored grey. S6 residues accessible to the central pore are colored green; S6 residues with intermediate accessibility are colored yellow; S6 residues not accessible to the central pore are colored red [77], [78]. (B) Extracellular view of the pore region of the cone channel in cartoon representation. The CNGA3 subunits are colored cyan (light and dark), and the CNGB3 subunits are colored orange (light and dark); the α-carbons of CNGA3 L361 and F385 and the corresponding CNGB3 R403 and F427 are shown as space-filled atoms. The distance between the α-carbons of residues in the same subunit, e.g., the distance between the α-carbons of L361 and F385 in the CNGA3 subunit, was 10 Å (marked by a black line in one of the subunits); the distance between the α-carbons of the residues in neighboring subunits, e.g., the distance between the α-carbons of CNGA3 F385 and CNGB3 R403, was 7 Å (marked by a blue line in one of the subunits). It is apparent that the residues can interact with each other [79].
(TIF)

Figure S3 The model structure of the cytosolic domain of the human cone channel is in agreement with experimental data. Side view of the CNBD tetramer in cartoon representation. The CNGA3 subunits are colored cyan (light and dark) and the CNGB3 subunits are colored orange (light and dark). The helices composing the C-linker, A′-F′, are marked; the helices of the CNBD, denoted A-C, are marked in one of the subunits. The inter-subunit interaction called “elbow on a shoulder” is evident: the “elbow” comprises the A′ and B′ helices, which rest on the “shoulder,” the C′ and D′ helices of the neighboring subunit. On the left, CNGA3 α-carbons of R436, E467 and D507 are shown as spheres; the distance between the R436 Cα and the Cα atoms of both E467 and D507 is 11 Å. Hence, R436 can form a salt bridge with E467 or D507, stabilizing the intra- and inter-subunit interface. On the right, the α-carbons of CNGA3 E467 and CNGB3 R478 and D549 are shown as spheres; the distance between the Cα of R436 and the α-carbons of the negatively charged residues is 11 Å. Therefore, R478 can form a salt bridge with E467 or D549, stabilizing the intra- or inter-subunit interface, respectively.
(TIF)

Figure S4 Molecular interpretations for selected disease-causing mutations. (A–D) The effects of the CNGA3 R427C mutation and the corresponding CNGB3 Y469D mutation. In each panel, CNBDs from two neighboring subunits are shown in side view. The α-carbons of CNGA3 R427 and E453, as well as of CNGB3 Y469, D488, and E495, are presented as spheres. The insets show a closer view of the residues' interactions, marked by black rectangles on the main panels. It is clear that CNGA3 R427 could interact with CNGA3 E453 (A) or CNGB3 D488 and E495 (D). Abolishment of the positive charge at position 427 by an R-to-C mutation may disrupt the inter-subunit electrostatic interactions. Similarly, the negative charge resulting from mutation of CNGB3 Y469 to D could result in repulsion of the mutated residue by the negatively charged CNGA3 E453 (C) or by CNGB3 D488 and E495 (B). The distances of the interacting residues are marked. (E) The effect of the CNGA3 L186F mutation. A side view of the TM region of the cone channel is presented; for clarity only one VSD is shown. CNGA3 L186, shown as a space-filling model, is located at the interface of the VSD with the p-loop. Replacement of L186 with a bulky Phe can interrupt the tight interface. (F) The effect of the CNGB3 S435F mutation. An intracellular view of the pore region of the cone channel is presented. S435 faces the central pore, and its replacement with a bulky Phe can disrupt the helix bundle and/or block the pore.
(TIF)

Figure S5 Contact maps of top-ranked predicted evolutionary couplings (ECs; red) overlaid on monomer (light grey) and intermonomer (dark grey) contacts from (A) the crystal structure of the bacterial MlotiK1 channel (TM domain); (B) the crystal structure of the mouse HCN2 channel (cytosolic domain). The blue and green lines show the location of α-helices and β-sheets in the sequence, respectively.
(TIF)

Figure S6 Detecting the TM helices of human CNGA3 (A) and CNGB3 (B) subunits. The locations of the TM helices according to secondary-structure prediction (PsiPred [63]), TM segment identification (MEMSAT [64] and HMMTOP [65]), profile-to-profile alignment (HHpred [66]) and fold recognition (FFAS03 [67]) are marked in different colors according to the legend. The boundaries of the TM helices that were used for the modeling are highlighted in yellow; the designation of TM helices is also marked and highlighted in yellow. The black dashed line shows the alternative location of helix S1. In (A) the grey lines show the corresponding boundaries of TM helices derived from the recent model-structure of the canine CNGA3 channel in open conformation [59]; some of the differences could be associated the conformational changes upon channel opening/closure. (C) The sequence alignment of the MlotK1, CNGA3 and CNGB3 channels used here for modeling the TM region. (D, E) Hydrophobicity profiles of the S1 (D) and S3 (E) regions among selected CNGA3 homologs. The sequences are colored according to the color-coding bar, with blue-through-yellow corresponding to hydrophilic-through-hydrophobic amino acids according to the Kessel/Ben-Tal hydrophobicity scale [76]. (D) Defining the boundaries of the S1 TM helix is complicated because of the low sequence similarity and high diversity among the hydrophobicity profiles of the sequences in this region. The red and black frames mark the final and alternative boundaries of the S1 helix, respectively. (E) The S3 region is much less challenging in that the various sequences manifest similar hydrophobicity profiles. The highly conserved Asp residue, marked by a red arrow, provides another clear signal for the alignment.
(TIF)

Figure S7 Mean-square displacement of the CNGA3 channel in the holo-state according to the GNM and ANM modes. (A) Motion I: The shape of GNM mode 1 fits the profile of ANM mode 3. (B) Motion II: the average shape of GNM modes 2-4 fits the profile of ANM mode 4. (C) Motion III: the average shape of GNM modes 5 and 6 fits the profile of ANM mode 12. The fluctuations of one chain are demonstrated, since the fluctuations of all four chains are identical. The locations of the VSD, the pore, the C-linker and the CNBD are marked on the x-axis. The hinge regions are marked by arrows and the corresponding residue numbers. The fluctuations of the channel in the apo-state according to GNM and ANM are nearly identical (not shown).
(TIF)

Figure S8 Elastic network analysis of the CNGA3 channel without the VSDs: Association of GNM and ANM modes. In each panel, chain A represents an arbitrary CNGA3 chain (the channel comprises four identical chains). (A, B) GNM mode 1 is associated with ANM mode 3. (A) Side view of the channel in cartoon representation colored according to the correlation of the S5 helix in chain A with the other residues in GNM mode 1. (B) Conformations of ANM mode 3, colored according to the direction of motion, ranging from green to red (side view). (C, D) GNM modes 2 and 3 are associated with ANM mode 4. (C) Side view of the channel in cartoon representation, colored according to the correlation of the chain A S5 with the other residues in GNM modes 2 and 3. (D) Conformations of ANM mode 4, colored according to the direction of motion, ranging from green to red (intracellular view). (A, C) The magnitude of positive and negative correlations between the fluctuations of the residues is color-coded according to the blue-to-red scale at the bottom of the picture. Positive correlation indicates motion of two residues in the same direction, while negative correlation indicates motion in opposite directions.
(TIF)

Figure S9 Pore-radius profiles as a function of the position along the membrane normal. The black curve shows the profile of the model structure of the (closed state of the) CNGA3 channel proposed here, and the green curve shows the profile of the edge conformation of the CNGA3 channel as predicted by the slowest ANM mode (rotational motion I). The profiles were calculated using the HOLE software [45]. Clearly, the pore is wider in the “rotated” conformation of the CNGA3 channel (green curve), consistent with the suggestion that rotational motion I is related to channel gating. For clarity, the pore region of two monomers is shown in ribbon representation, colored orange and blue. As expected, the narrowest regions in the pore correspond to the selectivity filter and the S6 helix bundle crossing.
(TIF)

Figure S10 Sequence alignment of mouse HCN2, CNGA3 and CNGB3 channels in the cytosolic region. The structural elements, α-helices (red lines) and β-sheets (blue arrows), are marked. The α-helices are labeled with capital letters; the β-sheets are numbered [21].
(TIF)

Figure S11 (A, B) The contribution of the 30 slowest GNM modes to the overall motion of the CNGA3 tetramer. The percentage of contribution was estimated as the weight of the frequency of a specific mode *n*, calculated considering the frequencies of all *N* modes (100λ_n_/Σλ_N_). (A) Holo-state. Modes 2 and 3 share the same eigenvalue, as do modes 5 and 6. We studied here the 6 slowest modes, each contributing over 4% to the overall motion of the channel. (B) Holo-state in the absence of the VSDs. Modes 2 and 3 share the same eigenvalue. We investigated the three slowest modes, each contributing over 3% to the overall motion of the channel. (C) Mean-square fluctuations of the CNGA3 tetramer in a holo-state in GNM modes 2–4. The shape of mode 4 (red) fits the profile of the average of modes 2 and 3 (black). The fluctuations of one chain of the homotetrameric channel are presented, since the fluctuations of the four chains are identical.
(TIF)

Table S1 Investigation of known disease-causing mutations in CNGA3. ConSurf grades were calculated using the ConSurf server [40], as described in the Methods section. The “Position occupancy in homologous proteins” column describes all possible amino acids featured in the corresponding positions in homologous sequences. Positions with a ConSurf grade of less than 5 are marked in bold. The “Effect” column describes the effect of the identified the mutation on channel function: deleterious refers to deleterious effect of the mutation on channel function; partially deleterious includes impaired or altered function of the mutated channel; n.d. – effect of the mutation was not determined. SF – selectivity filter.
(PDF)

Table S2 Investigation of disease-causing mutations in CNGB3. ConSurf grades were calculated using the ConSurf server [40], as described in the Methods section. The “Position occupancy in homologous proteins” column describes all possible amino acids featured in the corresponding positions in homologous sequences. None of the mutations has been examined experimentally.
(PDF)

Model S1 The model structure of the human cone channel comprising of two CNGA3 and two CNGB3 subunits.
(PDB)
