# Supplementary material for: Structure, Dynamics and Implied Gating Mechanism of a Human Cyclic Nucleotide-Gated Channel
Source: PLoS Comput Biol. 2014 Dec 4;10(12):e1003976. doi: 10.1371/journal.pcbi.1003976 (PMC4256070; doi:10.1371/journal.pcbi.1003976)
Supplement: Text S1 — The file contains the following sections: “Model structure is compatible with experimental data available for the homologous CNGA1 protein”, “Mapping disease-causing mutations on the model structure”, “Evolutionary couplings between amino acids that are not in contact in the model structure”, “Equilibrium dynamics of the CNGA3 channel” and “Supplementary references”. (PDF) [file pcbi.1003976.s001.pdf]

Model structure is compatible with experimental data available for the homologous CNGA1 protein

Much of the available data related to the CNG channels were collected from experiments on the bovine CNGA1 channel. Below we assess our model structure of the human cone channel with the applicable data from these experiments. The structure of the central pore of the bovine channel has been assessed through cysteine-accessibility analysis, using cysteine mutations and cysteine-specific modifications [1,2]. The available data on the accessibility of CNGA1 residues to the central pore were compatible with our model structure (Figure S2A).

Previous studies have shown interaction between two residues within a single subunit of the bovine channel; these residues correspond to L361 and F385 in CNGA3 and to R403 and F427 in CNGB3 [3]. In our model structure the α-carbons of CNGA3 L361 and F385 (or CNGB3 R403 and F427) from the same subunit were 11 Å apart; the α-carbons of CNGA3 L361 and F385 (or CNGB3 R403 and F427) from the neighboring subunits were 9 Å apart (Figure S2B). The distances between the residues indicate the possibility for both inter- and intra-subunit interactions.

The C-linker, connecting the TM domain and the CNBDs, is composed of helices marked A'-F' (Figure S3). In the resting state, these helices are arranged in a compact tetrameric structure so that the first two helices, A' and B', interact with helices C' and D' from the neighboring subunit (Figure S3). This structural motif is called "elbow on a shoulder": the "elbow", helices A' and B', rests on a "shoulder", helices C' and D' [4]. In the bovine channel, the interaction between the "elbow" and the "shoulder" is functionally important and is mediated by a triad of charged residues, stabilizing the closed state of the channel [5,6]. The triad corresponds to residues R436, E467 and D507 in CNGA3 and R478, A509 and D549 in CNGB3. At the

interface of the two CNGA3 subunits, the α-carbons of residues R436, E467 and D507 are 11 Å apart from each other (Figure S3). The model suggests that R436 could form a salt bridge with either E467 or D507 or both and stabilize inter-subunit and/or intra-subunit interaction. Similarly, at the interface of the CNGA3 and CNGB3 subunits, CNGB3 R478 could form a salt bridge with CNGA3 E467 or D549 (Figure S3). The proximity of these charged residues supports the model structure and suggests the residues' possible functional role in stabilization of the closed state. This role would be similar to that of the charged triad (R431, E462, D502) in the bovine channel [5,6].

Mapping disease-causing mutations on the model structure

We mapped all known disease-causing mutations of CNGA3 and CNGB3 onto the model structure (Tables S1 and S2; Figure S4). As anticipated, almost all known mutations, i.e., all 10 mutations in CNGB3 and 46 of 57 mutations in CNGA3, are found in evolutionarily conserved residues (ConSurf grades of 5 or larger), many of which are in the pore region and near the cGMP binding site. Of the 11 CNGA3 mutations found in more variable positions and shown in red bold in Table S1, four (E228K, T245M, S341P, E376K) have been found to affect trafficking and have a partial effect on channel function, two (D485V, E593K) have a partially deleterious effect on channel function, two (E194K, G267D) have a deleterious effect on channel function, and three (Y263D, N276S, G329C) have not been investigated experimentally (Table S1).

We also examined a subset of nine disease-associated mutations for which strong experimental data have been obtained in three or more independent studies. Two of these mutations are in the C-linker, and three are in the CNBD, including two that reside close to the cGMP binding site. The remaining four residues are in the VSD, including the conserved R277

and R283 (Table S1). This observation suggests an important evolutionarily selected role for the voltage-sensing motif, although the channel is voltage-insensitive.

Below we present three examples of possible molecular interpretations for the damage due to mutations associated with achromatopsia.

CNGA3 R427C/CNGB3 Y469D. The disease-causing mutation R427C in CNGA3 is of special interest because a mutation in the corresponding position in subunit CNGB3, i.e., Y469, is also disease-causing [7,8]. CNGA3 R427 is located in the A'-helix of the C-linker and can interact electrostatically with E453, located in the C'-helix of the adjacent CNGA3 subunit (Figure S4A). R427 can also interact with CNGB3 D488, located in the loop connecting helices B' and C' of the C-linker of the adjacent CNGB3 subunit, and with E495, located in helix C' of the adjacent CNGB3 subunit (Figure S4D). We suggest that interactions between these charged residues stabilize inter-subunit interfaces, as in the R431-E462-D502 triad in the bovine channel (Figure S3) [5,6]. The mutation of the positively charged R427 to Cys would abolish the electrostatic attraction to the negatively charged residues (CNGA3 E453 or CNGB3 D488 and E495; Figures S4A and S4D), disrupting the compact architecture of the C-linkers. The mutation of Y469 to the negatively charged Asp in CNGB3 (corresponds to CNGA3 R427) might repel D488 and E495 of the adjacent CNGB3 subunit (Figure S4B), as well as E453 of the adjacent CNGA3 subunit (Figure S4C). In this case, the architecture of the C-linkers would be disrupted due to the electrostatic repulsion between the negatively charged residues.

CNGA3 L186F. CNGA3 L186 is located in the S1 helix, at the interface with the S5 helix and the P-loop (Figure S4E). Clearly, the replacement of L186 with a bulky phenylalanine might disrupt this interface. Moreover, in voltage-gated channels this interface is functionally important [9]. Although CNG channels are not voltage-dependent, the role of the VSD in these channels is

unclear, and the interface between S1 and the pore might be still essential. Regardless of the functional role of the interface in CNG channels, its structure can be significantly disrupted by the L186F mutation.

CNGB3 S435F. CNGB3 S435 faces the central pore (Figure S4F), and its replacement with any large residue, e.g., phenylalanine, may disrupt the helix bundle, or simply block the pore. Indeed, CNGB3 homologs feature only small amino acids (S, A or G) in the position corresponding to S435, supporting the suggested interpretation of the effect of the S435F mutation.

Evolutionary couplings between amino acids that are not in contact in the model structure

In our analysis, most of the evolutionary couplings that did not correspond to actual amino acid contacts—more specifically, the majority of such couplings in the TM domain, and about half of such couplings in the cytosolic domain—had at least one residue in a loop region (data not shown). We hypothesized that these discrepancies emerged from differences in the conformation between CNGA3 and the bacterial template, and/or loop flexibility. To examine these possibilities, we calculated the evolutionary couplings of the templates directly (Figure S5). The overlay between the calculated evolutionary couplings and the contacts derived from the crystal structures (MlotiK1 channel and the cytosolic domain of mouse HCN2 channel) was similar to that of our model structure (Figures 3 and S5), and the majority of the observed false positive predictions had at least one residue in a loop region. Taken together, these observations indicate that many of the evolutionary couplings between pairs of amino acids that, according to the model structure, are not in direct contact with each other are likely to represent flexibility in the loop regions.

Equilibrium dynamics of the CNGA3 channel

A Gaussian network model (GNM) detected the dynamic domains of the channel and their cooperative motions. The six slowest GNM modes of motion emerged considerably above the rest in the eigenvalue spectrum (Figure S11A). These modes, therefore, represented the most important contributions to the overall motion of the channel, and we investigated them. We analyzed the shapes of the residues' mean-square displacement plots in the six slowest GNM modes. GNM modes 2 and 3, as well as GNM modes 5 and 6, shared the same eigenvalues (Figure S11A) and were degenerate. The average shape of the residues' fluctuations in modes 2 and 3 was very similar to mode 4, indicating that GNM modes 2–4 correspond to the same motion (Figure S11C). Thus, GNM modes 1–6 represented three types of motion. Next, we carried out anisotropic network model (ANM) analysis, which provided information on the directions of the motions in 3D-space. We associated the fluctuations (and correlations between fluctuations), detected by the GNM, to their directions, obtained from the ANM analysis, comparing the distributions of residue fluctuations in each ANM mode to the GNM modes (Figure S7). We further validated the modes' associations by mapping the GNM-derived cross-correlations between residues on the conformations obtained via ANM (Figure 4). Motion I was described by GNM mode 1 and ANM mode 3; motion II was described by GNM modes 2-4 and ANM mode 4; In motion III, described by GNM modes 5-6 and ANM mode 12 (Figures 4 and S7).

The CNG channel features VSDs but is, in essence, insensitive to the membrane potential. The role of the VSD is still unclear [10], although a recent study associated it with trafficking [11]. In order to understand the effect of the VSDs on channel dynamics, we performed GNM and ANM analysis of the CNGA3 channel (in holo-conformation), removing the VSDs. Three

GNM modes of motion emerged as the slowest in the eigenvalue spectrum (Figure S11B). GNM mode 1 was associated with ANM mode 3 (Figures S8A and S8B); this motion was very similar to motion I of the intact CNGA3 channel, in direction, mobility and cooperative dynamics (Figures 4A, 4B, S8A and S8B). GNM modes 2 and 3 were degenerate, and we related them to ANM mode 4 (Figures S8C and S8D). This motion resembled motion III of the intact channel, with some alterations in cooperativity dynamics (Figures 4E, 4F, S8C and S8D). Overall, the removal of the VSDs did not affect the slowest modes of motion of the CNGA3 channel, aside from the motions that directly involve the VSDs (motion II of the intact channel).

Supplementary references

1. Flynn GE, Zagotta WN (2001) Conformational changes in S6 coupled to the opening of cyclic nucleotide-gated channels. Neuron 30: 689-698.
2. Flynn GE, Zagotta WN (2003) A cysteine scan of the inner vestibule of cyclic nucleotide-gated channels reveals architecture and rearrangement of the pore. J Gen Physiol 121: 563-582.
3. Mazzolini M, Anselmi C, Torre V (2009) The analysis of desensitizing CNGA1 channels reveals molecular interactions essential for normal gating. J Gen Physiol 133: 375-386.
4. Zagotta WN, Olivier NB, Black KD, Young EC, Olson R, et al. (2003) Structural basis for modulation and agonist specificity of HCN pacemaker channels. Nature 425: 200-205.
5. Craven KB, Olivier NB, Zagotta WN (2008) C-terminal movement during gating in cyclic nucleotide-modulated channels. J Biol Chem 283: 14728-14738.
6. Craven KB, Zagotta WN (2004) Salt bridges and gating in the COOH-terminal region of HCN2 and CNGA1 channels. J Gen Physiol 124: 663-677.
7. Nishiguchi KM, Sandberg MA, Gorji N, Berson EL, Dryja TP (2005) Cone cGMP-gated channel mutations and clinical findings in patients with achromatopsia, macular degeneration, and other hereditary cone diseases. Hum Mutat 25: 248-258.
8. Wissinger B, Gamer D, Jagle H, Giorda R, Marx T, et al. (2001) CNGA3 mutations in hereditary cone photoreceptor disorders. Am J Hum Genet 69: 722-737.
9. Lee SY, Banerjee A, MacKinnon R (2009) Two separate interfaces between the voltage sensor and pore are required for the function of voltage-dependent K(+) channels. PLoS Biol 7: e47.
10. Clayton GM, Altieri S, Heginbotham L, Unger VM, Morais-Cabral JH (2008) Structure of the transmembrane regions of a bacterial cyclic nucleotide-regulated channel. Proc Natl Acad Sci USA 105: 1511-1515.
11. Faillace MP, Bernabeu RO, Korenbrot JI (2004) Cellular processing of cone photoreceptor cyclic GMP-gated ion channels: a role for the S4 structural motif. J Biol Chem 279: 22643-22653.
12. Ashkenazy H, Erez E, Martz E, Pupko T, Ben-Tal N (2010) ConSurf 2010: calculating evolutionary conservation in sequence and structure of proteins and nucleic acids. Nucleic Acids Res 38: W529-533.
13. Patel KA, Bartoli KM, Fandino RA, Ngatchou AN, Woch G, et al. (2005) Transmembrane S1 mutations in CNGA3 from achromatopsia 2 patients cause loss of function and impaired cellular trafficking of the cone CNG channel. Invest Ophthalmol Vis Sci 46: 2282-2290.
14. Muraki-Oda S, Toyoda F, Okada A, Tanabe S, Yamade S, et al. (2007) Functional analysis of rod monochromacy-associated missense mutations in the CNGA3 subunit of the cone photoreceptor cGMP-gated channel. Biochem Biophys Res Commun 362: 88-93.
15. Johnson S, Michaelides M, Aligianis IA, Ainsworth JR, Mollon JD, et al. (2004) Achromatopsia caused by novel mutations in both CNGA3 and CNGB3. J Med Genet 41: e20.
16. Reuter P, Koeppen K, Ladewig T, Kohl S, Baumann B, et al. (2008) Mutations in CNGA3 impair trafficking or function of cone cyclic nucleotide-gated channels, resulting in achromatopsia. Hum Mutat 29: 1228-1236.
17. Koeppen K, Reuter P, Kohl S, Baumann B, Ladewig T, et al. (2008) Functional analysis of human CNGA3 mutations associated with colour blindness suggests impaired surface expression of channel mutants A3(R427C) and A3(R563C). Eur J Neurosci 27: 2391-2401.

18. Azam M, Collin RW, Shah ST, Shah AA, Khan MI, et al. (2010) Novel CNGA3 and CNGB3 mutations in two Pakistani families with achromatopsia. Mol Vis 16: 774-781.
19. Saqib MA, Awan BM, Sarfraz M, Khan MN, Rashid S, et al. (2011) Genetic analysis of four Pakistani families with achromatopsia and a novel S4 motif mutation of CNGA3. Jpn J Ophthalmol 55: 676-680.
20. Liu C, Varnum MD (2005) Functional consequences of progressive cone dystrophy-associated mutations in the human cone photoreceptor cyclic nucleotide-gated channel CNGA3 subunit. Am J Physiol Cell Physiol 289: C187-198.
21. Ding XQ, Fitzgerald JB, Quiambao AB, Harry CS, Malykhina AP (2010) Molecular pathogenesis of achromatopsia associated with mutations in the cone cyclic nucleotide-gated channel CNGA3 subunit. Adv Exp Med Biol 664: 245-253.
22. Kohl S, Marx T, Giddings I, Jagle H, Jacobson SG, et al. (1998) Total colourblindness is caused by mutations in the gene encoding the alpha-subunit of the cone photoreceptor cGMP-gated cation channel. Nat Genet 19: 257-259.
23. Genead MA, Fishman GA, Rha J, Dubis AM, Bonci DM, et al. (2011) Photoreceptor structure and function in patients with congenital achromatopsia. Invest Ophthalmol Vis Sci 52: 7298-7308.
24. Vincent A, Wright T, Billingsley G, Westall C, Heon E (2011) Oligocone trichromacy is part of the spectrum of CNGA3-related cone system disorders. Ophthalmic Genet 32: 107-113.
25. Koeppen K, Reuter P, Ladewig T, Kohl S, Baumann B, et al. (2010) Dissecting the pathogenic mechanisms of mutations in the pore region of the human cone photoreceptor cyclic nucleotide-gated channel. Hum Mutat 31: 830-839.
26. Trankner D, Jagle H, Kohl S, Apfelstedt-Sylla E, Sharpe LT, et al. (2004) Molecular basis of an inherited form of incomplete achromatopsia. J Neurosci 24: 138-147.
27. Ahuja Y, Kohl S, Traboulsi EI (2008) CNGA3 mutations in two United Arab Emirates families with achromatopsia. Mol Vis 14: 1293-1297.
28. Wang X, Wang H, Cao M, Li Z, Chen X, et al. (2011) Whole-exome sequencing identifies ALMS1, IQCB1, CNGA3, and MYO7A mutations in patients with Leber congenital amaurosis. Hum Mutat 32: 1450-1459.
29. Duricka DL, Brown RL, Varnum MD (2012) Defective trafficking of cone photoreceptor CNG channels induces the unfolded protein response and ER-stress-associated cell death. Biochem J 441: 685-696.
30. Kohl S, Varsanyi B, Antunes GA, Baumann B, Hoyng CB, et al. (2005) CNGB3 mutations account for 50% of all cases with autosomal recessive achromatopsia. Eur J Hum Genet 13: 302-308.
31. Kohl S, Baumann B, Broghammer M, Jagle H, Sieving P, et al. (2000) Mutations in the CNGB3 gene encoding the beta-subunit of the cone photoreceptor cGMP-gated channel are responsible for achromatopsia (ACHM3) linked to chromosome 8q21. Hum Mol Genet 9: 2107-2116.
