# Supplementary figures and images for: Structure, Dynamics and Implied Gating Mechanism of a Human Cyclic Nucleotide-Gated Channel
Source: PLoS Comput Biol. 2014 Dec 4;10(12):e1003976. doi: 10.1371/journal.pcbi.1003976 (PMC4256070; doi:10.1371/journal.pcbi.1003976)

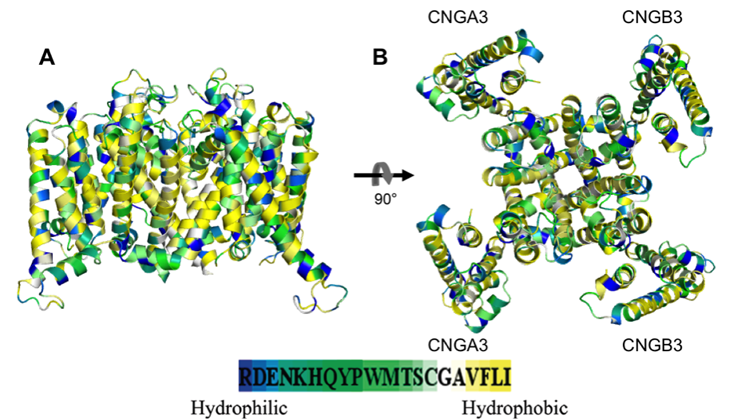

Supplement: Figure S1 — The hydrophobicity pattern of the model structure of the TM domain of the human cone channel from side (A) and extracellular (B) views. The model structure is colored according to the color-coding bar, with blue-through-yellow corresponding to hydrophilic-through-hydrophobic amino acids according to the Kessel/Ben-Tal hydrophobicity scale [76]. The structure displays a typical hydrophobicity pattern, with hydrophobic residues exposed to the lipid, and charged and polar amino acids buried in the protein core or located in the extramembrane regions. (TIF) [file pcbi.1003976.s002.tif]

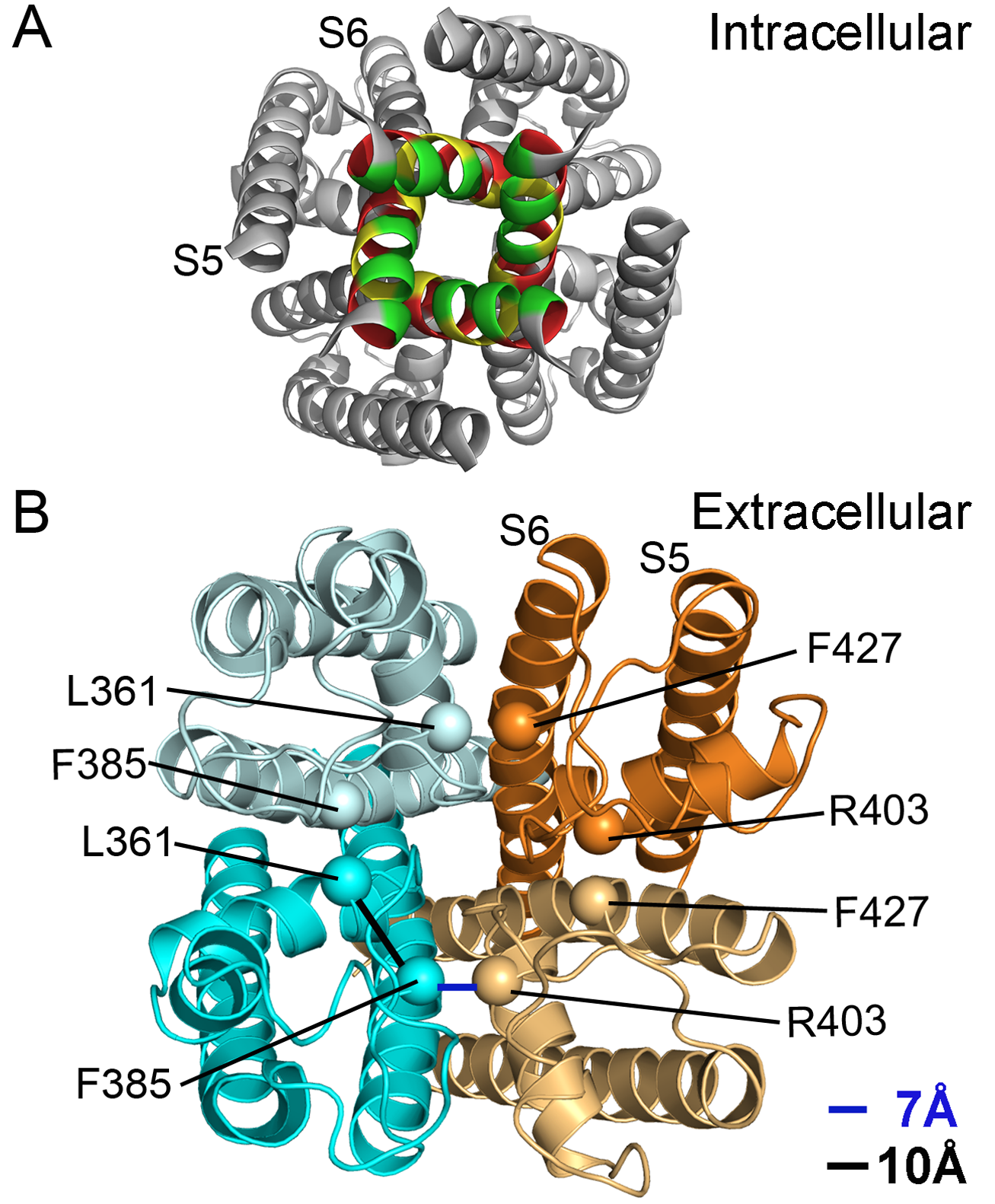

Supplement: Figure S2 — The model structure of the TM domain of the human cone channel is in agreement with experimental data. (A) Intracellular view of the pore region of the channel in cartoon representation. The channel is colored grey. S6 residues accessible to the central pore are colored green; S6 residues with intermediate accessibility are colored yellow; S6 residues not accessible to the central pore are colored red [77], [78]. (B) Extracellular view of the pore region of the cone channel in cartoon representation. The CNGA3 subunits are colored cyan (light and dark), and the CNGB3 subunits are colored orange (light and dark); the α-carbons of CNGA3 L361 and F385 and the corresponding CNGB3 R403 and F427 are shown as space-filled atoms. The distance between the α-carbons of residues in the same subunit, e.g., the distance between the α-carbons of L361 and F385 in the CNGA3 subunit, was 10 Å (marked by a black line in one of the subunits); the distance between the α-carbons of the residues in neighboring subunits, e.g., the distance between the α-carbons of CNGA3 F385 and CNGB3 R403, was 7 Å (marked by a blue line in one of the subunits). It is apparent that the residues can interact with each other [79]. (TIF) [file pcbi.1003976.s003.tif]

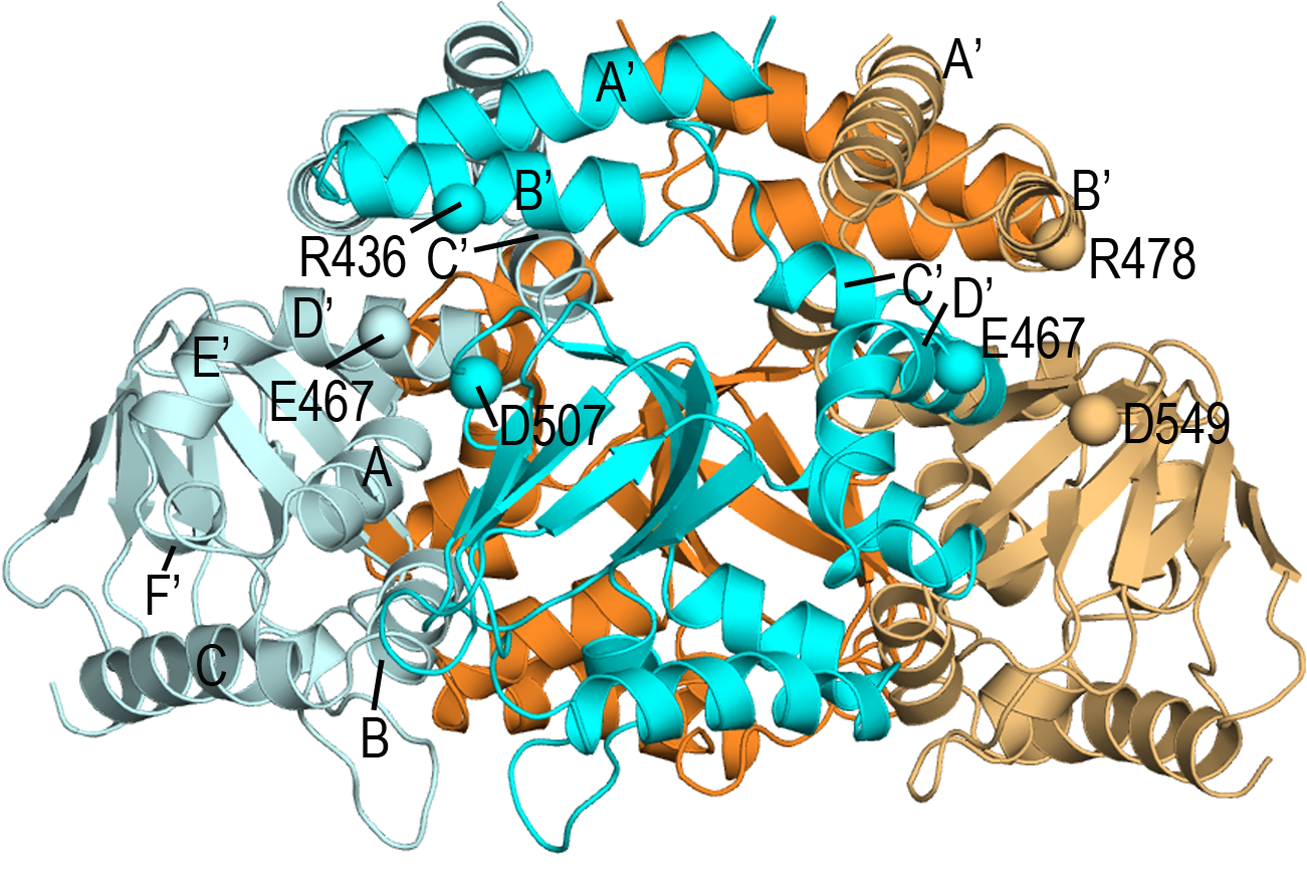

Supplement: Figure S3 — The model structure of the cytosolic domain of the human cone channel is in agreement with experimental data. Side view of the CNBD tetramer in cartoon representation. The CNGA3 subunits are colored cyan (light and dark) and the CNGB3 subunits are colored orange (light and dark). The helices composing the C-linker, A′-F′, are marked; the helices of the CNBD, denoted A-C, are marked in one of the subunits. The inter-subunit interaction called “elbow on a shoulder” is evident: the “elbow” comprises the A′ and B′ helices, which rest on the “shoulder,” the C′ and D′ helices of the neighboring subunit. On the left, CNGA3 α-carbons of R436, E467 and D507 are shown as spheres; the distance between the R436 Cα and the Cα atoms of both E467 and D507 is 11 Å. Hence, R436 can form a salt bridge with E467 or D507, stabilizing the intra- and inter-subunit interface. On the right, the α-carbons of CNGA3 E467 and CNGB3 R478 and D549 are shown as spheres; the distance between the Cα of R436 and the α-carbons of the negatively charged residues is 11 Å. Therefore, R478 can form a salt bridge with E467 or D549, stabilizing the intra- or inter-subunit interface, respectively. (TIF) [file pcbi.1003976.s004.tif]

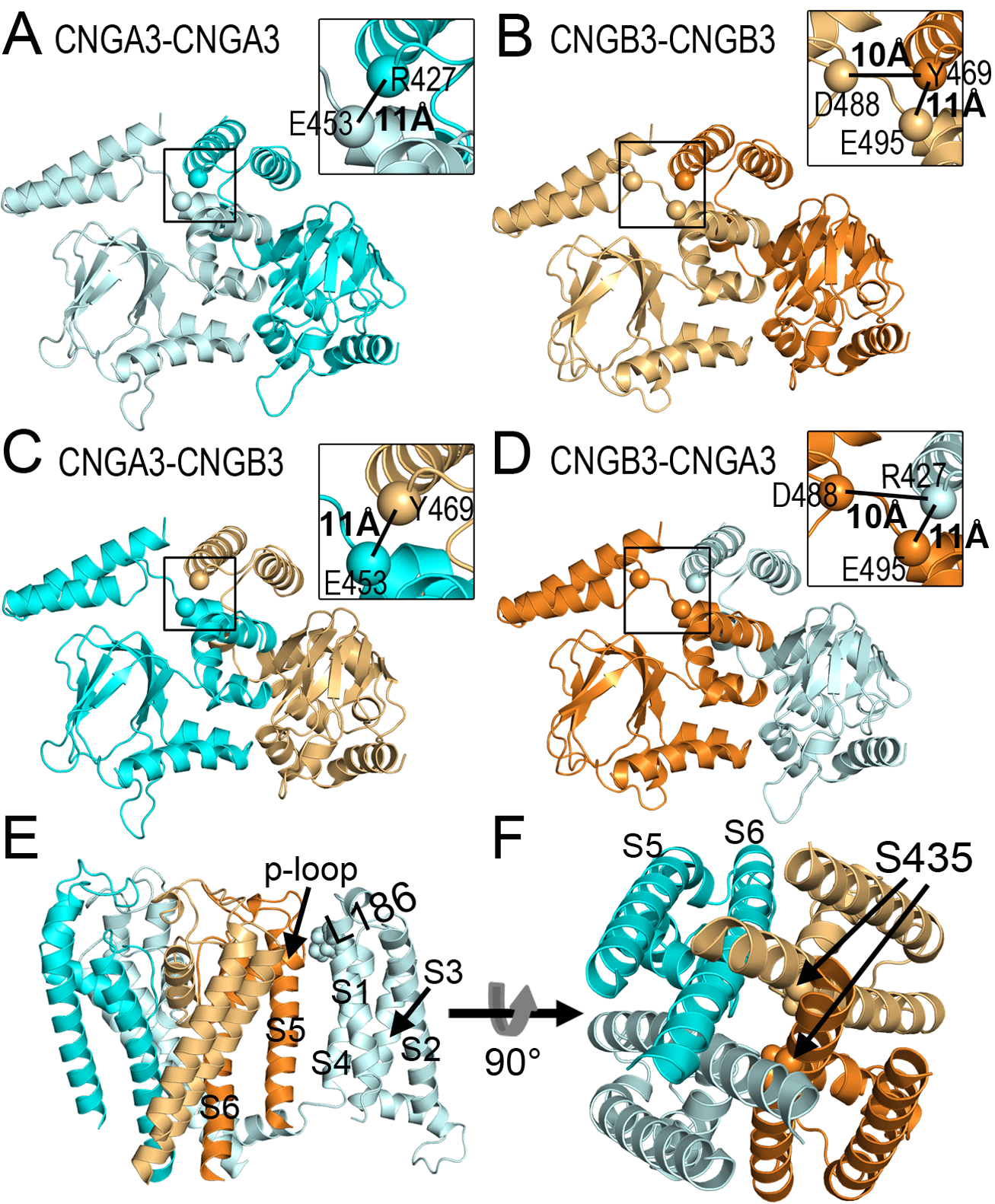

Supplement: Figure S4 — Molecular interpretations for selected disease-causing mutations. (A–D) The effects of the CNGA3 R427C mutation and the corresponding CNGB3 Y469D mutation. In each panel, CNBDs from two neighboring subunits are shown in side view. The α-carbons of CNGA3 R427 and E453, as well as of CNGB3 Y469, D488, and E495, are presented as spheres. The insets show a closer view of the residues' interactions, marked by black rectangles on the main panels. It is clear that CNGA3 R427 could interact with CNGA3 E453 (A) or CNGB3 D488 and E495 (D). Abolishment of the positive charge at position 427 by an R-to-C mutation may disrupt the inter-subunit electrostatic interactions. Similarly, the negative charge resulting from mutation of CNGB3 Y469 to D could result in repulsion of the mutated residue by the negatively charged CNGA3 E453 (C) or by CNGB3 D488 and E495 (B). The distances of the interacting residues are marked. (E) The effect of the CNGA3 L186F mutation. A side view of the TM region of the cone channel is presented; for clarity only one VSD is shown. CNGA3 L186, shown as a space-filling model, is located at the interface of the VSD with the p-loop. Replacement of L186 with a bulky Phe can interrupt the tight interface. (F) The effect of the CNGB3 S435F mutation. An intracellular view of the pore region of the cone channel is presented. S435 faces the central pore, and its replacement with a bulky Phe can disrupt the helix bundle and/or block the pore. (TIF) [file pcbi.1003976.s005.tif]

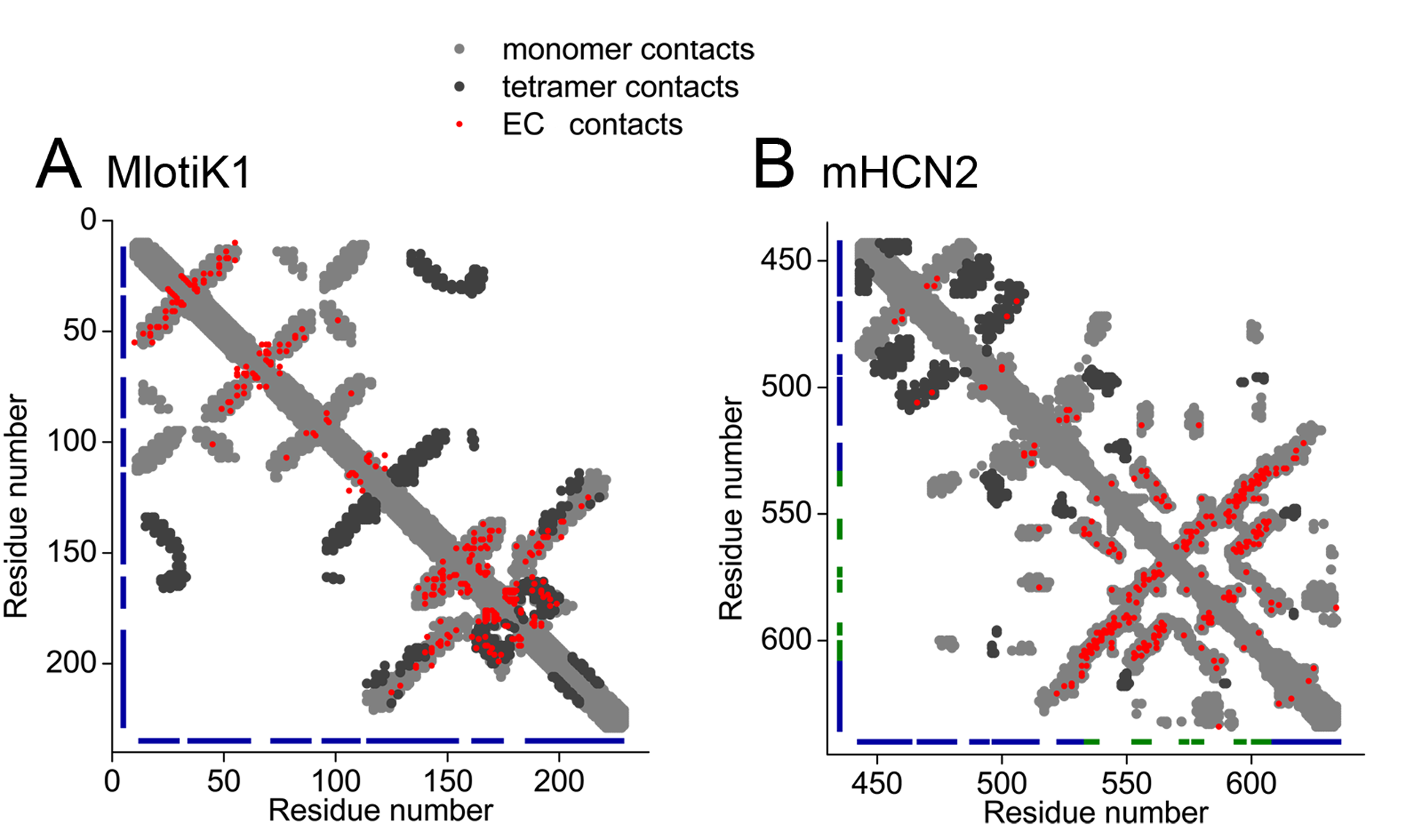

Supplement: Figure S5 — Contact maps of top-ranked predicted evolutionary couplings (ECs; red) overlaid on monomer (light grey) and intermonomer (dark grey) contacts from (A) the crystal structure of the bacterial MlotiK1 channel (TM domain); (B) the crystal structure of the mouse HCN2 channel (cytosolic domain). The blue and green lines show the location of α-helices and β-sheets in the sequence, respectively. (TIF) [file pcbi.1003976.s006.tif]

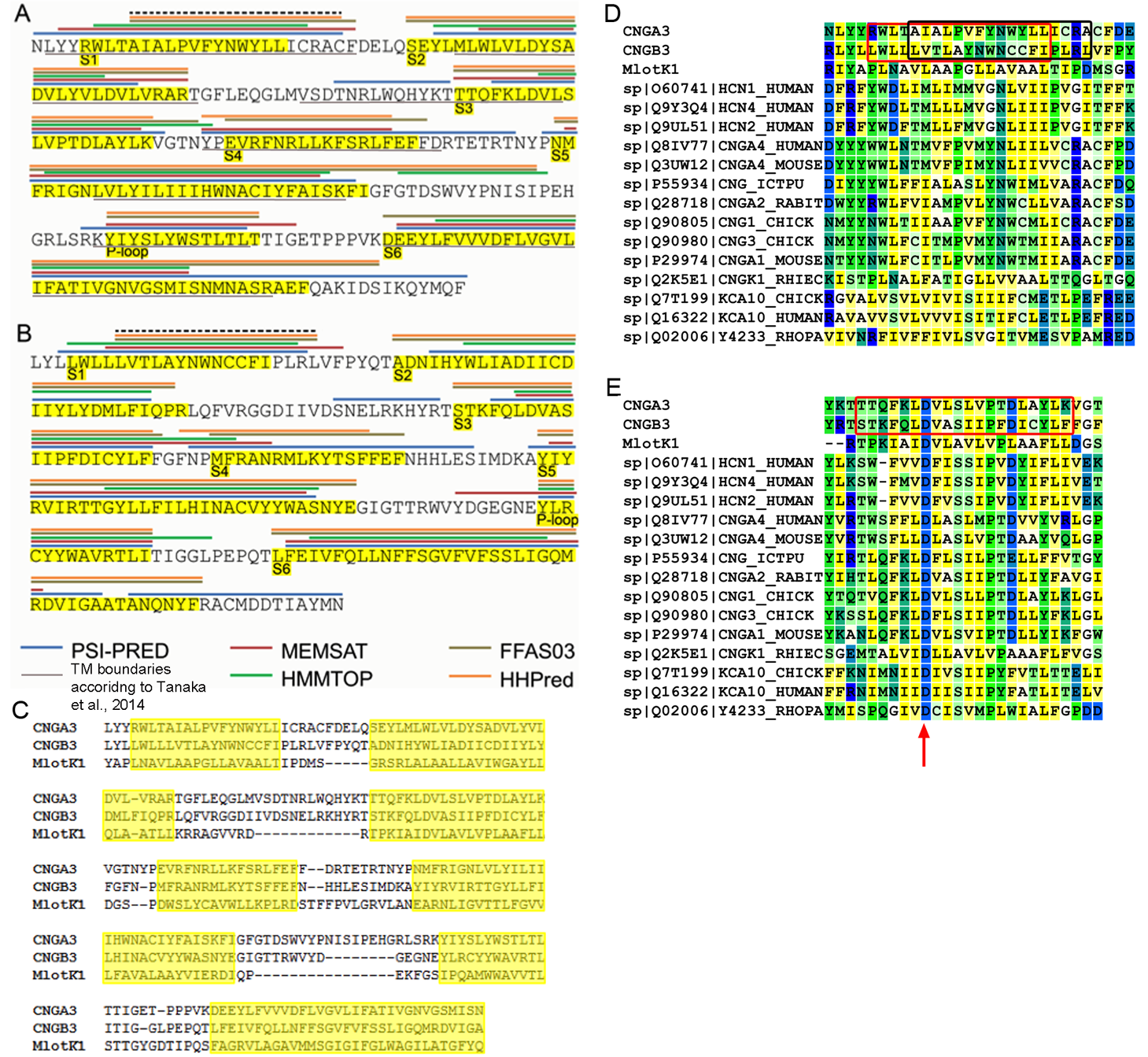

Supplement: Figure S6 — Detecting the TM helices of human CNGA3 (A) and CNGB3 (B) subunits. The locations of the TM helices according to secondary-structure prediction (PsiPred [63]), TM segment identification (MEMSAT [64] and HMMTOP [65]), profile-to-profile alignment (HHpred [66]) and fold recognition (FFAS03 [67]) are marked in different colors according to the legend. The boundaries of the TM helices that were used for the modeling are highlighted in yellow; the designation of TM helices is also marked and highlighted in yellow. The black dashed line shows the alternative location of helix S1. In (A) the grey lines show the corresponding boundaries of TM helices derived from the recent model-structure of the canine CNGA3 channel in open conformation [59]; some of the differences could be associated the conformational changes upon channel opening/closure. (C) The sequence alignment of the MlotK1, CNGA3 and CNGB3 channels used here for modeling the TM region. (D, E) Hydrophobicity profiles of the S1 (D) and S3 (E) regions among selected CNGA3 homologs. The sequences are colored according to the color-coding bar, with blue-through-yellow corresponding to hydrophilic-through-hydrophobic amino acids according to the Kessel/Ben-Tal hydrophobicity scale [76]. (D) Defining the boundaries of the S1 TM helix is complicated because of the low sequence similarity and high diversity among the hydrophobicity profiles of the sequences in this region. The red and black frames mark the final and alternative boundaries of the S1 helix, respectively. (E) The S3 region is much less challenging in that the various sequences manifest similar hydrophobicity profiles. The highly conserved Asp residue, marked by a red arrow, provides another clear signal for the alignment. (TIF) [file pcbi.1003976.s007.tif]

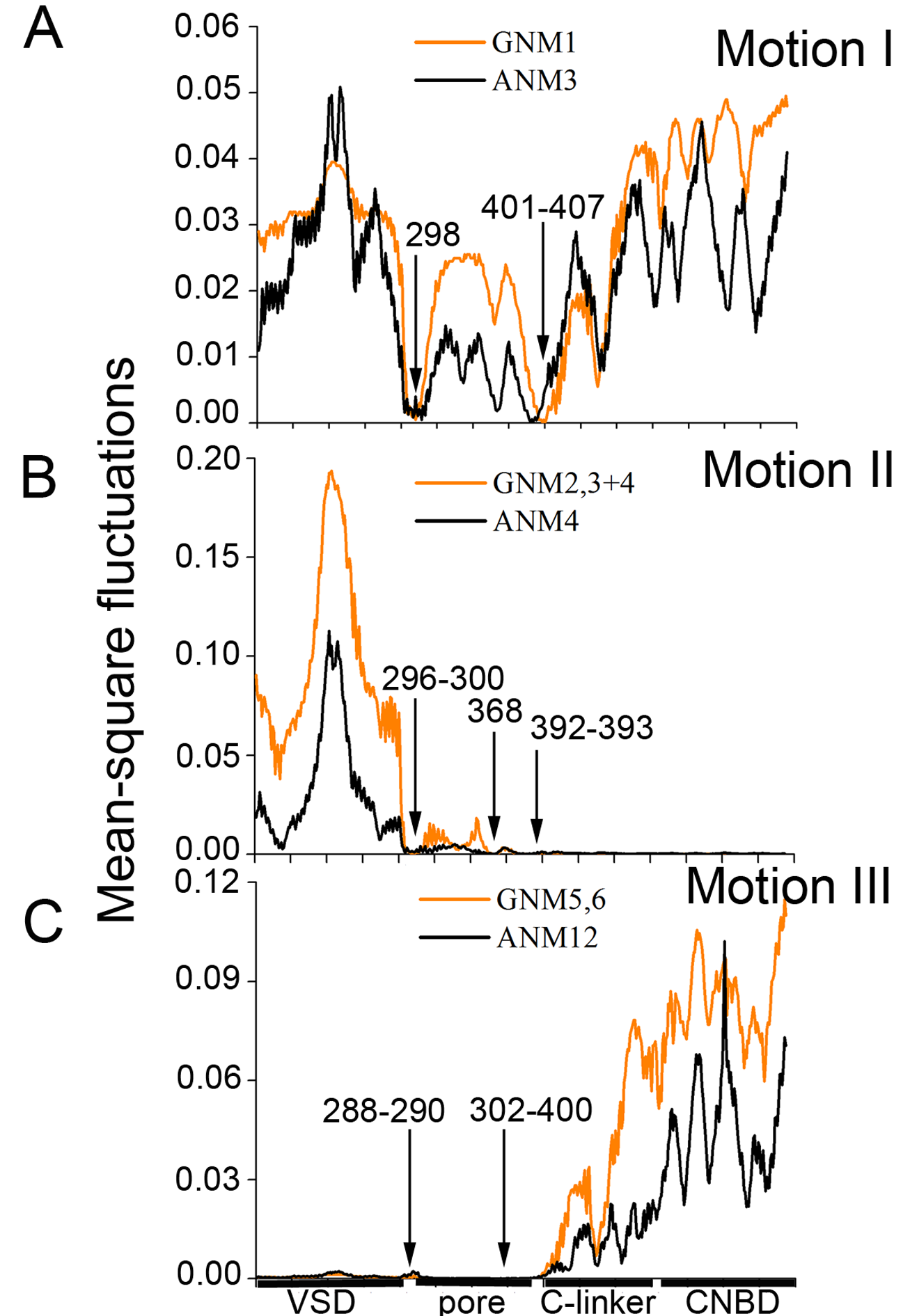

Supplement: Figure S7 — Mean-square displacement of the CNGA3 channel in the holo-state according to the GNM and ANM modes. (A) Motion I: The shape of GNM mode 1 fits the profile of ANM mode 3. (B) Motion II: the average shape of GNM modes 2-4 fits the profile of ANM mode 4. (C) Motion III: the average shape of GNM modes 5 and 6 fits the profile of ANM mode 12. The fluctuations of one chain are demonstrated, since the fluctuations of all four chains are identical. The locations of the VSD, the pore, the C-linker and the CNBD are marked on the x-axis. The hinge regions are marked by arrows and the corresponding residue numbers. The fluctuations of the channel in the apo-state according to GNM and ANM are nearly identical (not shown). (TIF) [file pcbi.1003976.s008.tif]

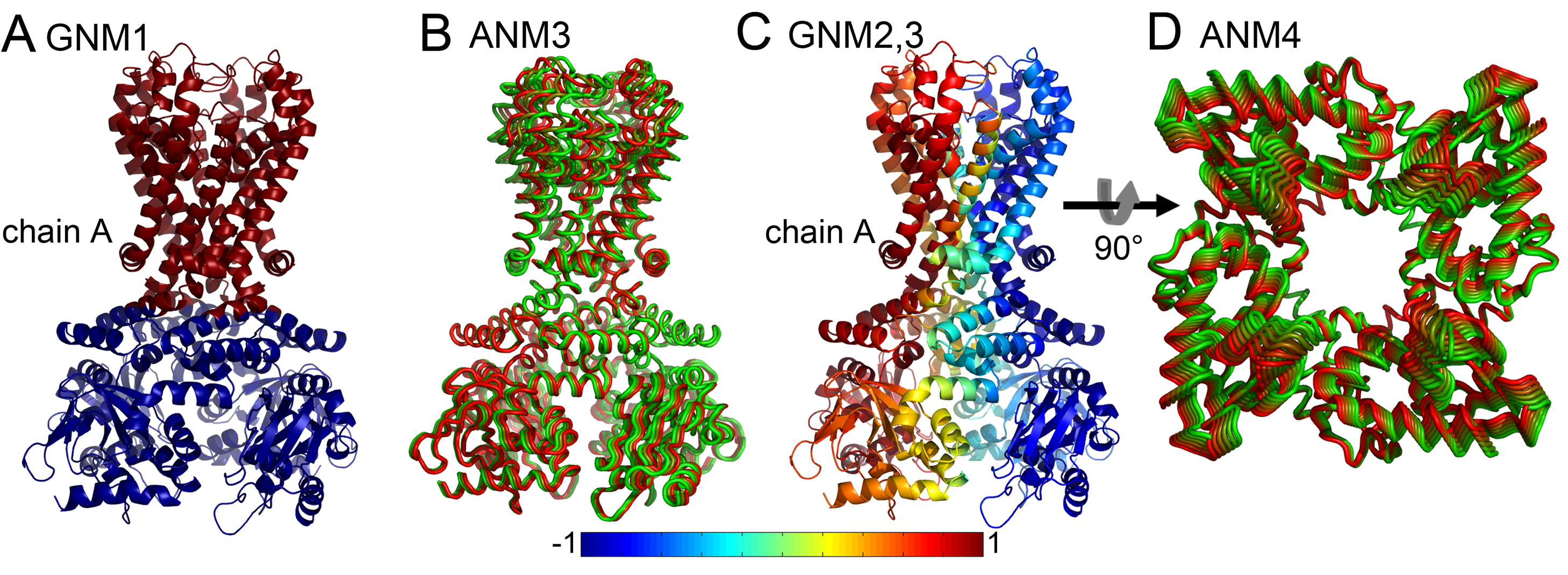

Supplement: Figure S8 — Elastic network analysis of the CNGA3 channel without the VSDs: Association of GNM and ANM modes. In each panel, chain A represents an arbitrary CNGA3 chain (the channel comprises four identical chains). (A, B) GNM mode 1 is associated with ANM mode 3. (A) Side view of the channel in cartoon representation colored according to the correlation of the S5 helix in chain A with the other residues in GNM mode 1. (B) Conformations of ANM mode 3, colored according to the direction of motion, ranging from green to red (side view). (C, D) GNM modes 2 and 3 are associated with ANM mode 4. (C) Side view of the channel in cartoon representation, colored according to the correlation of the chain A S5 with the other residues in GNM modes 2 and 3. (D) Conformations of ANM mode 4, colored according to the direction of motion, ranging from green to red (intracellular view). (A, C) The magnitude of positive and negative correlations between the fluctuations of the residues is color-coded according to the blue-to-red scale at the bottom of the picture. Positive correlation indicates motion of two residues in the same direction, while negative correlation indicates motion in opposite directions. (TIF) [file pcbi.1003976.s009.tif]

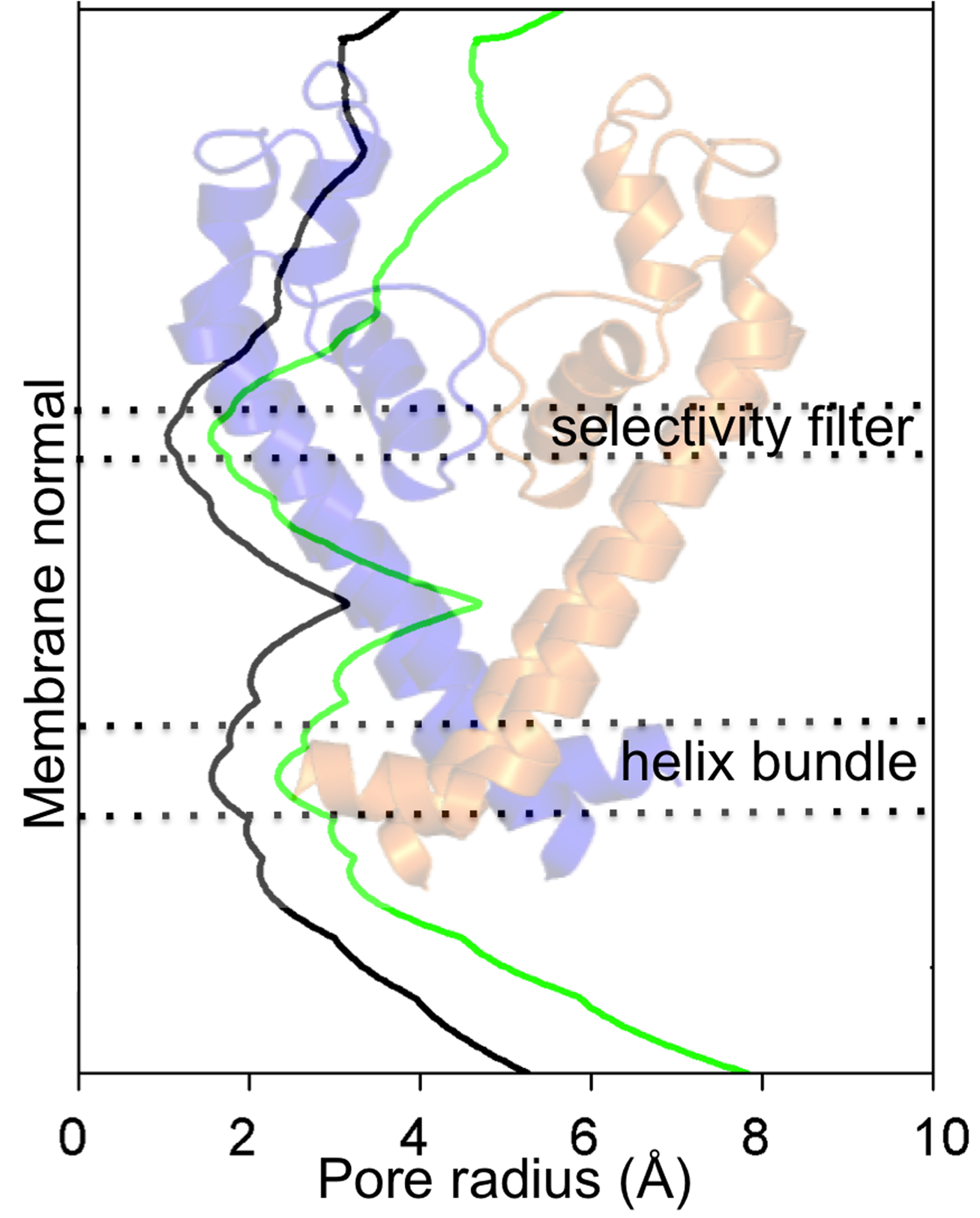

Supplement: Figure S9 — Pore-radius profiles as a function of the position along the membrane normal. The black curve shows the profile of the model structure of the (closed state of the) CNGA3 channel proposed here, and the green curve shows the profile of the edge conformation of the CNGA3 channel as predicted by the slowest ANM mode (rotational motion I). The profiles were calculated using the HOLE software [45]. Clearly, the pore is wider in the “rotated” conformation of the CNGA3 channel (green curve), consistent with the suggestion that rotational motion I is related to channel gating. For clarity, the pore region of two monomers is shown in ribbon representation, colored orange and blue. As expected, the narrowest regions in the pore correspond to the selectivity filter and the S6 helix bundle crossing. (TIF) [file pcbi.1003976.s010.tif]

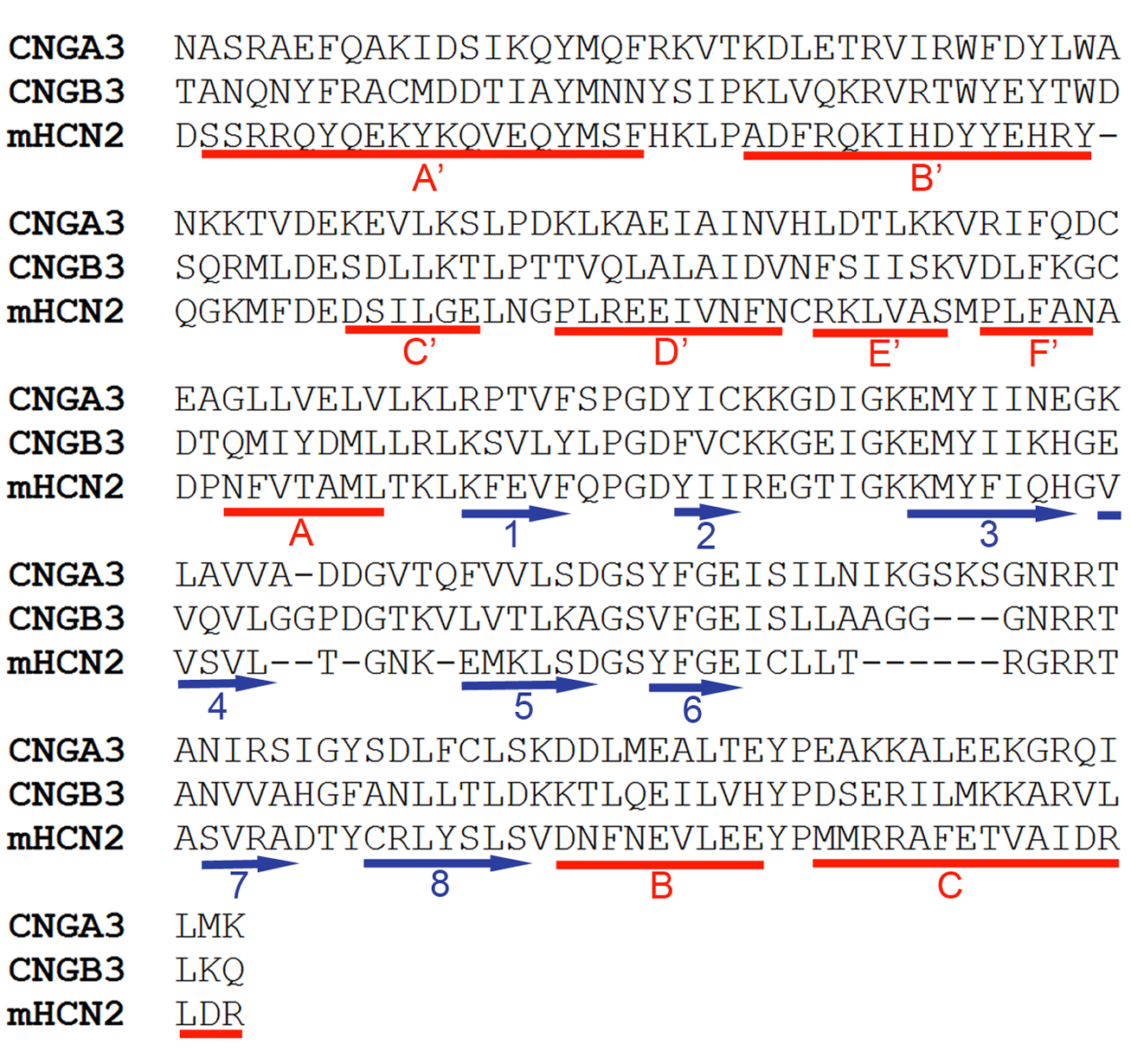

Supplement: Figure S10 — Sequence alignment of mouse HCN2, CNGA3 and CNGB3 channels in the cytosolic region. The structural elements, α-helices (red lines) and β-sheets (blue arrows), are marked. The α-helices are labeled with capital letters; the β-sheets are numbered [21]. (TIF) [file pcbi.1003976.s011.tif]

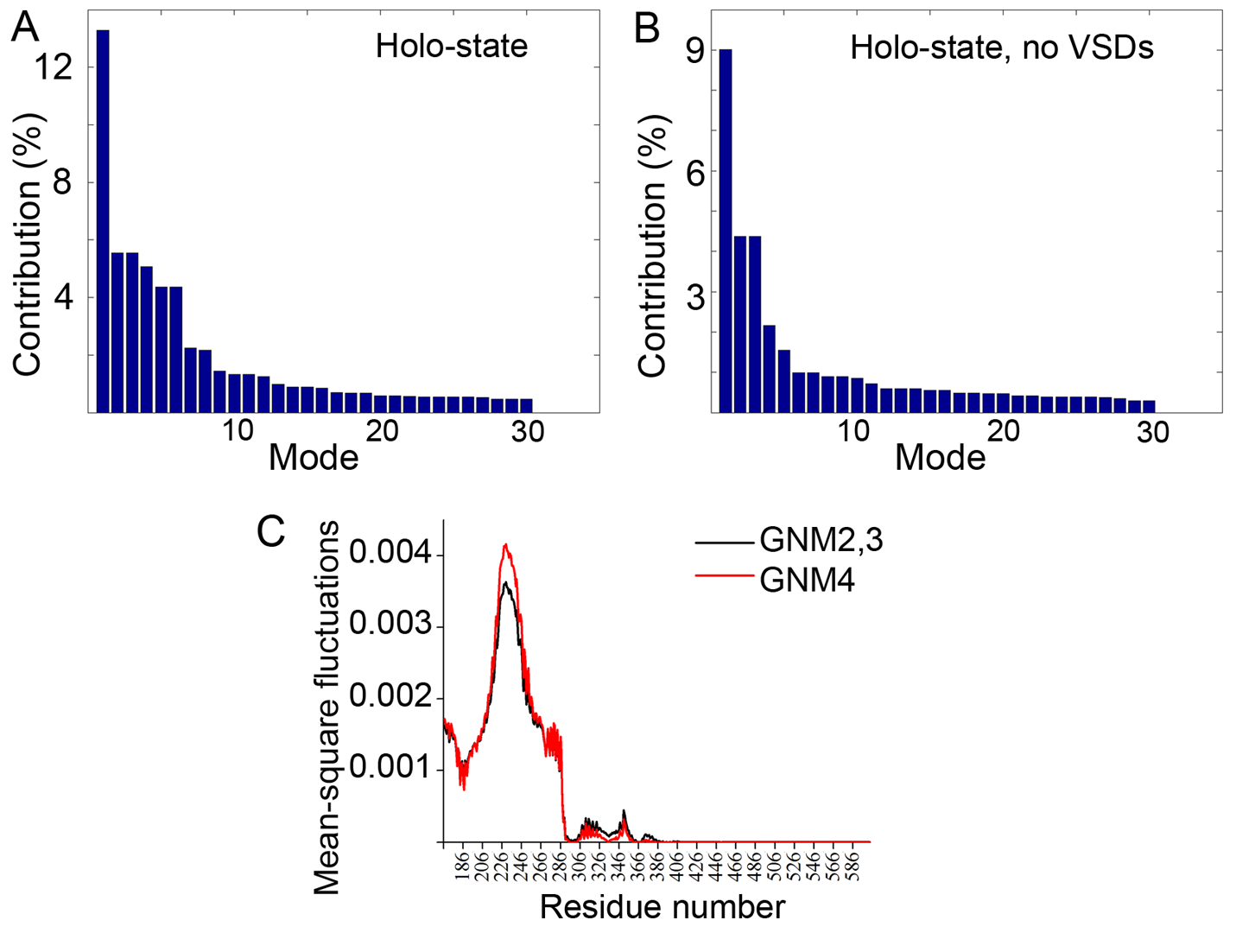

Supplement: Figure S11 — (A, B) The contribution of the 30 slowest GNM modes to the overall motion of the CNGA3 tetramer. The percentage of contribution was estimated as the weight of the frequency of a specific mode n, calculated considering the frequencies of all N modes (100λn/ΣλN). (A) Holo-state. Modes 2 and 3 share the same eigenvalue, as do modes 5 and 6. We studied here the 6 slowest modes, each contributing over 4% to the overall motion of the channel. (B) Holo-state in the absence of the VSDs. Modes 2 and 3 share the same eigenvalue. We investigated the three slowest modes, each contributing over 3% to the overall motion of the channel. (C) Mean-square fluctuations of the CNGA3 tetramer in a holo-state in GNM modes 2–4. The shape of mode 4 (red) fits the profile of the average of modes 2 and 3 (black). The fluctuations of one chain of the homotetrameric channel are presented, since the fluctuations of the four chains are identical. (TIF) [file pcbi.1003976.s012.tif]
