# Supplementary material for: Structure, Dynamics and Implied Gating Mechanism of a Human Cyclic Nucleotide-Gated Channel
Source: PLoS Comput Biol. 2014 Dec 4;10(12):e1003976. doi: 10.1371/journal.pcbi.1003976 (PMC4256070; doi:10.1371/journal.pcbi.1003976)
Supplement: Table S1 — Investigation of known disease-causing mutations in CNGA3. ConSurf grades were calculated using the ConSurf server [40], as described in the Methods section. The “Position occupancy in homologous proteins” column describes all possible amino acids featured in the corresponding positions in homologous sequences. Positions with a ConSurf grade of less than 5 are marked in bold. The “Effect” column describes the effect of the identified the mutation on channel function: deleterious refers to deleterious effect of the mutation on channel function; partially deleterious includes impaired or altered function of the mutated channel; n.d. – effect of the mutation was not determined. SF – selectivity filter. (PDF) [file pcbi.1003976.s013.pdf]

Table S1. Investigation of known disease-causing mutations in CNGA3. ConSurf grades were calculated using the ConSurf server [12], as described in the Methods section. The "Position occupancy in homologous proteins" column describes all possible amino acids featured in the corresponding positions in homologous sequences. Positions with a ConSurf grade of less than 5 are marked in **bold**. The "Effect" column describes the effect of the identified the mutation on channel function: deleterious refers to deleterious effect of the mutation on channel function; partially deleterious includes impaired or altered function of the mutated channel; n.d. – effect of the mutation was not determined. SF – selectivity filter.

Mutation	ConSurf grade	Location in the model structure	Position occupancy in homologous proteins	Effect
Y181C	7	S1	A,W,M,I,G,Y,V	Deleterious+impaired trafficking [8,13,14]
N182Y	8	S1	H,Q,T,N,I,V	Deleterious+impaired trafficking [8,13]
L186F	6	S1	T,C,I,L,V	Deleterious+impaired trafficking [8,13]
C191Y	6	Loop S1-S2	F,S,A,T,C,G,V	Partially deleterious+impaired trafficking [8,13]
**E194K**	2	Loop S1-S2	F,S,T,N,K,Y,E,V,Q,C,L,A,P,H,D,R	Deleterious [8,14]
R223W	8	S2/Loop S2-S3	F,H,N,I,R	Deleterious [7,8,14,15]
T224R	8	S2/Loop S2-S3	S,M,T,K,I,E,L,V	n.d. [8]
**E228K**	4	Loop S2-S3	F,S,T,N,K,E,V,H,Q,D,I,R,G,L	Partially deleterious+Impaired trafficking [16]
**T245M**	1	Loop S2-S3	F,A,S,T,N,K,Y,V,H,Q,M,D,R,G,L	Partially deleterious+Impaired trafficking [15]
F249S	8	S3	S,A,F,W,L	n.d. [7]
D252N	9	S3	H,N,D	Deleterious [17]
D260N	8	S3	S,D,G,Y,L,E	Deleterious [8,14]
**Y263D**	4	S3	A,F,N,Y,H,Q,M,C,I,L	n.d. [7]
**G267D**	3	S3-S4 loop	F,S,N,K,P,E,V,H,Q,D,I,G,L	Deleterious [8,14]
R274S	8	S4	H,S,Q,T,P,R,G	n.d. [18]
**N276S**	4	S4	F,S,T,N,P,V,I,R,G,L	n.d. [19]
R277C/H	8	S4	Q,D,K,R	Deleterious+impaired trafficking [7,8,14,20,21]
R283Q/W	8	S4	A,S,T,P,K,Q,C,R	Deleterious+Impaired trafficking [8,16,21,22]
T291R	5	S4-S5 linker	F,S,W,T,P,K,V,M,R,I,L	Partially deleterious+Impaired trafficking [8,16,22]
**G329C**	3	Loop S5-P-loop	F,A,S,T,N,K,P,E,V,D,I,G,L	n.d. [23]
**S341P**	1	Loop S5-P-loop	F,S,T,N,K,Y,E,V,M,L,A,P,D,I,R,G	Partially deleterious+Impaired trafficking [7,8,14,17]
Y357C	9	P-loop	A,F,W,Y	n.d. [24]
L363P	8	SF	A,M,T,I,L	Deleterious [25]
G367V	9	SF	G	Partially deleterious+Impaired trafficking [25]

Mutation	ConSurf grade	Location in the model structure	Position occupancy in homologous proteins	Experimental verification
T369S	7	SF	S,A,T,N,K,V,H,Q,M,D,R,L	Partially deleterious [8,26]
P372S	8	Loop P-loop-S6	S,A,P	Deleterious+Impaired trafficking [8,14,17]
**E376K**	1	S6	F,A,S,T,N,P,Y,E,V,H,D,C,I,G,L	Partially deleterious+Impaired trafficking [25]
F380S	8	S6	F,M,I,L,Y	Deleterious [8,14]
G397V	9	S6	A,T,G	n.d. [27]
S401P	7	S6	A,S,T,N,D,E	n.d. [7]
M406T	7	S6	S,A,E,V,M,R,I,L	Partially deleterious [8,14]
R410W	7	A'-helix	S,A,T,K,E,H,Q,D,R,G	Deleterious [7,8,14,22]
R427C	7	A'-helix	F,N,K,Y,H,Q,M,R,L	Partially deleterious+Impaired trafficking [7,8,14,17]
L433W	5	B'-helix	F,T,M,I,L,V	Deleterious [17]
R436W	8	B'-helix	Q,K,R,G,E	Deleterious+Impaired trafficking [7,8,14,15]
R439W	5	B'-helix	S,T,K,E,H,Q,M,D,R,L	Partially deleterious+Impaired trafficking [16]
A469T	7	D'-helix	S,A,T,K,Y,V,C,D,I,G,L	Partially deleterious [16]
N471S	6	D'-helix	A,F,S,N,E,Y,H,Q,D	Partially deleterious [8,14,20]
**D485V**	4	F'-helix	S,A,T,N,K,E,H,Q,D,G,L	Partially deleterious [8,14]
C510S	8	β-roll	F,C,I,L,Y,V	Deleterious [8,14]
G513E	9	β-roll	H,N,G,E	Deleterious [8,14]
D514V	7	β-roll	A,S,T,N,E,H,Q,D	n.d. [23]
G516E	8	β-roll	A,I,G,L,V	Deleterious [8,14]
I522T	9	β-roll	I,V	Deleterious [8,14]
G525D	9	β-roll	G	Deleterious [8,14]
L527M	8	β-roll	F,A,M,I,L,V	n.d. [8,28]
V529M	9	β-roll	A,I,E,L,V	Deleterious [7,8,14,22]
F547L	9	β-roll	F,I,L,V	Partially deleterious+Impaired trafficking [8,15,16,22]
G548R	9	β-roll	D,G	Impaired trafficking [15,16]
G557R	7	β-roll	S,Q,G	Partially deleterious+Impaired trafficking [16,22]
R563H/C	8	β-roll	H,Q,P,K,R,L,V	Partially deleterious+Impaired trafficking [8,14,17,20,29]
T565M	8	β-roll	A,S,T,N,V,H,M,C,R,I	Partially deleterious [7,8]
R569H	7	β-roll	H,T,I,R,K,L,V	Deleterious [8,14,15]
S570I	9	β-roll	A,S,T,C,G	n.d. [23]

Mutation	ConSurf grade	Location in the model structure	Position occupancy in homologous proteins	Experimental verification
Y573C	7	β-roll	F,T,N,Y,V,H,C,D	Deleterious [8,14]
E590K	5	β-roll	S,F,N,Y,E,V,H,Q,M,D,I,R,L	Partially deleterious+Impaired trafficking [7,16]
**E593K**	3	C-helix	A,T,N,E,V,H,Q,M,D,I,R,L	Partially deleterious [8,14]
