# Supplementary material for: Structure, Dynamics and Implied Gating Mechanism of a Human Cyclic Nucleotide-Gated Channel
Source: PLoS Comput Biol. 2014 Dec 4;10(12):e1003976. doi: 10.1371/journal.pcbi.1003976 (PMC4256070; doi:10.1371/journal.pcbi.1003976)
Supplement: Table S2 — Investigation of disease-causing mutations in CNGB3. ConSurf grades were calculated using the ConSurf server [40], as described in the Methods section. The “Position occupancy in homologous proteins” column describes all possible amino acids featured in the corresponding positions in homologous sequences. None of the mutations has been examined experimentally. (PDF) [file pcbi.1003976.s014.pdf]

Table S2. Investigation of disease-causing mutations in CNGB3. ConSurf grades were calculated using the ConSurf server [12], as described in the Methods section. The "Position occupancy in homologous proteins" column describes all possible amino acids featured in the corresponding positions in homologous sequences. None of the mutations has been examined experimentally.

Mutation	ConSurf grade	Location in the model structure	Position occupancy in homologous proteins	Reference
P309L	9	S3	P	[30]
R403Q	7	P-loop	F,A,S,T,K,V,D,C,I,R,L	[7,30]
S435F	9	S6	S,A,G,V	[31]
M466T	8	Helix A'	S,A,V,M,C,I,G,L	[7]
Y469D	7	Helix A'	F,A,N,K,Y,H,Q,M,C,R,L	[7]
D494N	7	Loop B'-C'	F,Q,T,N,D,R,K,G,E	[7]
D513Y	5	Helix D'	F,S,T,N,K,Y,E,H,Q,C,D,L	[7]
F525N	8	Helix F'	F,L,V	[15]
G558C	7	cGMP binding region	A,T,I,P,G,L,V	[7]
L595F	7	cGMP binding region	F,A,S,W,N,Y,V,C,I,L	[7]
